# Natural Polyphenols Inhibit the Dimerization of the SARS-CoV-2 Main Protease: The Case of Fortunellin and Its Structural Analogs

**DOI:** 10.3390/molecules26196068

**Published:** 2021-10-07

**Authors:** Athanasios A. Panagiotopoulos, Ioannis Karakasiliotis, Danai-Maria Kotzampasi, Marios Dimitriou, George Sourvinos, Marilena Kampa, Stergios Pirintsos, Elias Castanas, Vangelis Daskalakis

**Affiliations:** 1 Laboratory of Experimental Endocrinology, School of Medicine, University of Crete, 71003 Heraklion, Greece; athpanagiotopoulos@hotmail.com (A.A.P.); med7p1120043@med.uoc.gr (D.-M.K.); kampam@uoc.gr (M.K.); 2 Laboratory of Biology, School of Medicine, Democritus University of Thrace, 68100 Alexandroupolis, Greece; ioakarak@med.duth.gr (I.K.); mardimitriou7@gmail.com (M.D.); 3 Laboratory of Virology, School of Medicine, University of Crete, 71003 Heraklion, Greece; sourving@uoc.gr; 4 Nature Crete Pharmaceuticals, 71305 Heraklion, Greece; pirintsos@uoc.gr; 5 Department of Biology, University of Crete, 71409 Heraklion, Greece; 6 Botanical Garden, University of Crete, 74100 Rethymnon, Greece; 7 Department of Chemical Engineering, Cyprus University of Technology, 3603 Limassol, Cyprus

**Keywords:** SARS-CoV-2, COVID-19, molecular simulations, metadynamics, natural products

## Abstract

3CL-Pro is the SARS-CoV-2 main protease (MPro). It acts as a homodimer to cleave the large polyprotein 1ab transcript into proteins that are necessary for viral growth and replication. 3CL-Pro has been one of the most studied SARS-CoV-2 proteins and a main target of therapeutics. A number of drug candidates have been reported, including natural products. Here, we employ elaborate computational methods to explore the dimerization of the 3CL-Pro protein, and we formulate a computational context to identify potential inhibitors of this process. We report that fortunellin (acacetin 7-*O*-neohesperidoside), a natural flavonoid *O*-glycoside, and its structural analogs are potent inhibitors of 3CL-Pro dimerization, inhibiting viral plaque formation in vitro. We thus propose a novel basis for the search of pharmaceuticals as well as dietary supplements in the fight against SARS-CoV-2 and COVID-19.

## 1. Introduction

Severe acute respiratory syndrome coronavirus 2 (SARS-CoV-2) was first identified in December 2019 and is the causative agent of coronavirus disease 2019 (COVID-19). It became a global pandemic, threatening the lives of millions of people belonging to vulnerable health groups. Intense scientific efforts worldwide resulted in the identification of the SARS-CoV-2 genomic structure, the viral protein sequence, and the structure and disease characteristics [1,2]. The main goal of the scientific community is to understand the molecular basis of the virus infection and replication along with the identification of potential drug targets [3]. The combined efforts include structural biophysics and computational modeling for resolving the key features of the viral infection at atomistic detail. The dynamic aspect of the SARS-CoV-2 proteins are crucial as well.

3CL-Pro (*3*-*chymotrypsin*-*like protease*) is the main protease of SARS-CoV-2 (MPro) and plays a vital role in cleaving the large polyprotein 1ab (replicase 1ab, ~790 kDa), translated by the virus RNA, at 11 different sites and liberating proteins indispensable for viral replication and proliferation with a unique specificity not found in any human protease [4]. Therefore, it represents a preferential target for the development of a series of non-toxic inhibitors against viral replication [5]. The individual monomers of SARS-CoV-2 3CL-Pro are inactive despite the fact that the active site resides within only one monomer of the active homodimer [6]. The latter has been the target of numerous anti-coronaviral drugs from peptides to small molecules [3,7,8,9] and thus, inhibitors of its dimerization are in need. Each 3CL-Pro monomer consists of fewer than 310 residues [10], while the dimerization is enabled by the interaction between the 3CL-Pro monomers that ensure its catalytic activity due to dimerization-induced conformational changes within the active site [10].

The chemical space of natural products, and especially that of polyphenols, has provided valuable candidate lead molecules in a number of diseases, including COVID-19 [5,11,12]. All-atom molecular dynamics (MD) simulations, along with enhanced sampling techniques and elaborate methods of analysis, have allowed unprecedented insight into complex phenomena in biology at extreme efficiency and accuracy, especially for the conformational changes of proteins [13,14,15]. A comprehensive review on antiviral agents and simulation approaches employed for SARS-CoV-2 proteins can be found in [3].

Here, we have explored the conformational space and dynamics of SARS-CoV-2 3CL-Pro by elaborate MD sampling methods that target not only the protein-ligand binding stabilization commonly explored in the literature [3,16] but the key implications for its dimerization. We have identified potential candidates that can inhibit its dimerization. Fortunellin (acacetin 7-*O*-neohesperidoside), a natural flavonoid *O*-glycoside isolated from the fruits of *Citrus japonica* var. *margarita* (kumquat) [17], was found as a lead compound that, together with a series of 16 structurally related analogs including apiin and rhoifolin, could be used as the basis for the design of novel antiviral compounds or dietary supplements to support the fight against COVID-19.

## 2. Materials and Methods

The Swiss Model Biospace contains 1628 entries of 3CL-Pro (September 2021), bound or not to ligands, and deposited in the Protein Data Bank (https://www.rcsb.org/, accessed on 30 September 2021) with high structural similarity (List S1 in the Supplementary Materials). Classical MD studies were based on two different crystals (pdb codes: 6YB7, 6LU7) [18] in order to increase the phase space sampling. Classical MD simulations of the fully solvated 3CL-Pro monomer with the atom interactions described by the Amber03 force field were performed in GROMACS 2020 [19]. Twenty trajectories, 3.0μs long each, were generated for the monomer (60 μs total) starting at the two different crystals and with varying salt content (see Supplementary Materials for details). Retrieved protein trajectories were further analyzed using Markov state modeling (MSM) [20]. Time-structure independent components analysis (tICA) was used to reduce the dimensionality of our data in PyEMMA [21]. MSM gave the conformational phase space of 3CL-Pro in two collective variables (termed CV-1/CV-2) determined by the torsional angles of residues 3, 4, 5, 6, 84, 135, 141, 164, 167, 171, 175, 178, 179, 180, 190, 195, 217, 284, 285, 286, 290, 291, 300, and 301. The classical MD runs on the 3CL-Pro monomer were used only for identifying the important residues for the protein conformational space.

Docking simulations of natural products, along with their analogs, from the ZINC database (http://zinc.docking.org/, accessed on 30 September 2021) [22] were performed on the 3CL-Pro crystal structure (pdb: 6YB7) as well as on key conformations out of the classical MD trajectories of the monomer produced by clustering in a fully flexible manner [23]. Fortunellin was identified as a key compound.

Based on the docked poses of the 3CL-Pro monomer/natural product complex, we initiated classical MD trajectories of the 3CL-Pro homodimer in the absence and the presence of fortunellin (10 μs), which was followed by 10 μs of enhanced sampling MD simulations on the 3CL-Pro homodimer in the absence of any ligand (parallel tempering metadynamics at the well-tempered ensemble, PTmetaD-WTE) [24]. Nine replicas were employed for the PTmetaD-WTE run at temperatures between 310–400 K (see Supplementary Materials for details). The extensive sampling in trajectories of classical MD (60 μs for the monomer and +10 μs for the homodimer) was necessary in order to generate the required large number of conformations for MSM validity [25]. Due to the large system size, extensive sampling is also important for the enhanced sampling methods to reach convergence for the associated free energy surfaces (FES) of the probed configurational space of 3CL-Pro [26]. In fact, we observed that the associated FES does not change after 8 μs of sampling at the PTmetaD-WTE level is reached.

A detailed description of the computational methods used in this study and the functions that describe the conformational space of 3CL-Pro based on the MSM analysis can be found in the Supplementary Material.

In vitro assays were performed as described in a recent publication from our group [27]. Briefly, SARS-CoV-2 (isolate 30-287) was obtained through cultures in Vero E6 cells (ATCC^®^ CRL-1586) from an infected patient in Alexandroupolis, Greece. Virus stock was prepared by infecting fully confluent Vero E6 cells in DMEM, supplemented with 10% fetal bovine serum (FBS), antibiotics at 37 °C, and 5% CO_2_. Infections were carried out in 96-well plates using SARS-CoV-2 (m.o.i. of 0.1) on Vero E6 cells. Cells were treated with different concentrations of fortunellin (ranging from 10^−10^ to 10^−6^ M, purchased from Biosynth Carbosynth, Bratislava, SVK) in a volume of 15 μL per 150 μL of medium for 48 h. Cell morphology was observed with phase contrast in an inverted microscope to record plaque formation. Microscopic images taken with the same microscope settings were analyzed with the Fiji software package (https://fiji.sc/, accessed on 30 September 2021).

For Western blot analysis, whole SARS-CoV-2-transfected Vero E6 cell extracts, treated or not (control) with 10^−6^ M fortunellin, were loaded at 30 μg per lane onto a 12.5% nondenaturing polyacrylamide gel for fractionation [28]. Proteins were transferred to nitrocellulose membranes and immunostained overnight using anti-SARS-CoV-2 3CL-Pro (rabbit polyclonal antibody, PA5-116940, Invitrogen, Waltham, MA, USA) at a dilution of 1:1000 at 4 °C. Peroxidase-conjugated anti-rabbit secondary antibodies (mouse, IgG-HRP, sc-2357, Santa Cruz Biotechnology, Dallas, TX, USA) were used for 1 h at room temperature. Chemiluminescence was determined using the ECL detection system (Luminata^TM^ Forte Western HRP substrate, Lot No. 173563, Merck, Darmstadt, Germany), according to the manufacturer’s protocol.

## 3. Results and Discussion

### 3.1. CL-Pro and Natural Product Docking: The Case of Fortunellin

**Figure 1:**
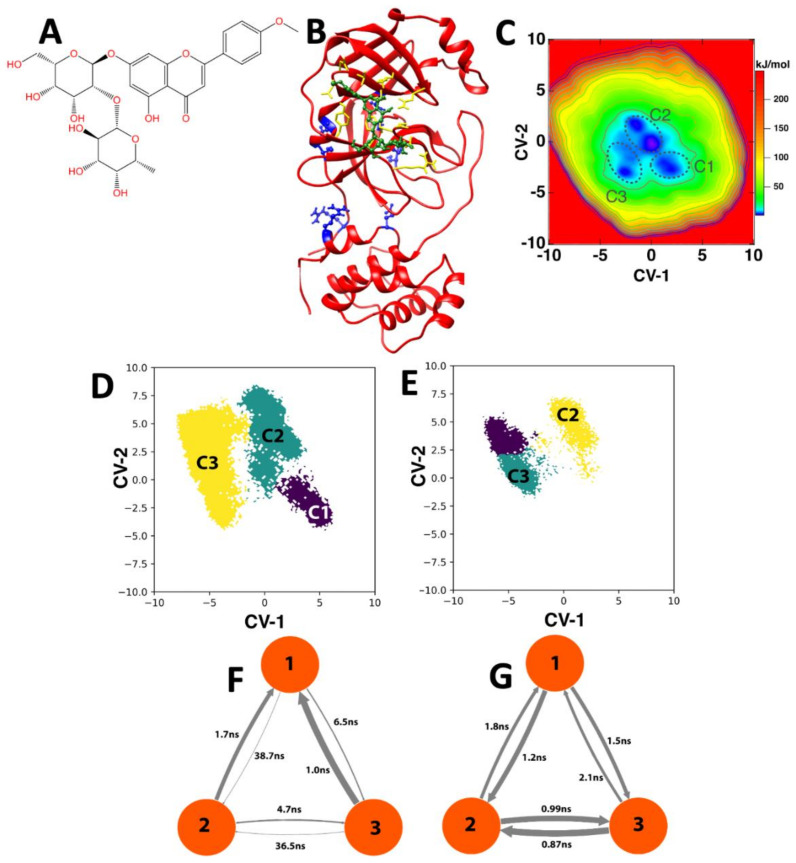
(**A**). The molecular structure of fortunellin. (**B**). The interaction of fortunellin with the 3CL-Pro monomer. Fortunellin is shown in green, the interacting amino acids are shown in yellow, and the dimerization interacting amino acids are shown in blue. (**C**). The free energy surfaces (FES) from the PTmetaD-WTE-enhanced sampling runs. Three minima of the 3CL-Pro homodimer dynamics are identified at the blue regions (C1, C2, and C3) on the CV–1/CV–2 phase space. (**D**). The identified states of 3CL-Pro in the absence of fortunellin within CV–1/CV–2 phase space. (**E**). The identified states of 3CL-Pro in the presence of fortunellin within the refined CV–1/CV–2 phase space. The C1–C3 minima are indicated on the D–E graphs based on the comparison with Figure 1A. (**F**,**G**). The transition times between the C1 and C3 minima are calculated in the absence (**F**) and in the presence (**G**) of fortunellin.

We utilized the FTMap server [29] to identify binding hot spots, determine drugability, and provide information about fragment-based drug discovery on the 3CL-Pro. The results of the FTMap permitted us to design a minimal structure of a potential 3CL-Pro dimerization inhibitor. Interrogating the ZINC database of natural products with this minimal structure, we identified *fortunellin* (*Acacetin*-*7*-*O*-*neohesperidoside*, ZINC4349204, Figure 1A) as a potential natural inhibitor of 3CL-Pro dimerization. The binding of fortunellin on the 3CL-Pro monomer (Figure 1B) revealed a high affinity binding (ΔG −13.9 kcal/mol) in a fully flexible, in silico binding and strong interactions with 3CL-Pro amino acids (Leu_32_, Asp_33_, Asp_34_, Val_35_, Tyr_37_, Gln_83_, Lys_88_, Tyr_101_, Lys_102_, Phe_103_, Val_104_, Arg_105_, Asp_108_, Phe_159_, Cys_160_, Asp_176_, Leu_177_, and Glu_178_). Despite the fact that fortunellin does not bind at the dimerization interface as defined by the residues 4, 10, 11, 14, 28, 139, 140, 147, 290, and 298, the binding may allosterically inhibit or weaken the formation of the active homodimer (see below). To increase the statistical significance of the docking studies, we also used the different poses of the monomeric 3CL-Pro MD trajectories (60 μs) at 1 ns intervals (poses were retrieved every 3 μs) as scaffolds for the fortunellin binding. As shown, the affinity of fortunellin fluctuated around −12.3 kcal/mol (SD ±1.005 kcal/mol), and after initial fluctuations and preceding significant changes of the dimerization domain as expressed by changes of the local RMSD value as compared to the crystal structure of the 3CL-Pro, it stabilized after 30 μs (see Supplementary Material Table S2).

### 3.2. Molecular Dynamics Simulations

A 10 μs enhanced sampling at the PTmetaD-WTE level [24] in the absence of a ligand was based on the refined CV phase space of key torsional angles of 3CL-Pro residues (see Methods and Supplementary Material) and revealed three main 3CL-Pro conformations (Figure 1C) at the minima of the produced free energy surface (FES). The first conformation (C1) corresponds to the crystal structure of the homodimer (inter-monomer distance at 1.72–1.78 Å), while the other two (C2-C3) correspond to the homodimers with higher distances between the monomers (1.86–1.93 Å), resulting in weakened inter-monomer interactions. The inter-monomer distances were calculated based on the minimum distances between residues on the native inter-monomer interface (4, 10, 11, 14, 28, 139, 140, 147, 290, and 298) as proposed in [30].

In Figure 1D,E, we present the MSM analysis of the equilibrium dynamics of the 3CL-Pro homodimer in the presence and absence of fortunellin (10 μs), respectively, on the MSM-refined CV phase space (CV-1/CV-2). The C1–C3 minima are assigned in these graphs based on a structural feature comparison with Figure 1C. Based on the MSM, we calculated that the transitions between the different states (C1–C3) occur at the average time scales of 37.6 ns (slow processes) and 3.5 ns (fast processes) in the absence of fortunellin (Figure 1F). However, the average transition time scale in the presence of fortunellin dropped to 1.42 ns with only fast transitions (Figure 1G). This significantly smaller time interval resulted in transitions between energy minima faster when in the presence of fortunellin, blocking the trapping at certain states and inhibiting the formation of the homodimer by sampling other non-favorable monomer conformations accessible at an ambient temperature, i.e., with greater distance between monomers that favor its dissociation. We noted that the C1 minimum that is associated with the crystal structure (active for dimerization) in Figure 1D is absent in Figure 1E, indicating that fortunellin disfavors this state. Instead, C1 was replaced by an alternative protein state, which is indicated by the purple area in Figure 1E.

### 3.3. CL-Pro Mutations Do Not Have a Direct Effect on the Fortunellin Binding or the Associated Inhibition of 3CL-Pro Dimerization

The 3CL-Pro C44-P52 loop has been proposed to host mutations but only at the T45, S46, E47, and L50 positions [31]. The latter is not listed as important residues in the 3CL-Pro/fortunellin dynamics analyzed by the MSM (see Supplementary Material). This gives us confidence that fortunellin can target 3CL-Pro even when 3CL-Pro is mutated. It appears, therefore, that the natural polyphenol fortunellin is a good drug (or dietary supplement) candidate for combatting COVID-19. The bulk of the scientific effort targeting 3CL-Pro focuses on the discovery or repurposing of the inhibitors of its enzymatic activity [32]. Here, we have instead directed our efforts toward the identification of (natural) compounds, which could inhibit the dimerization of the enzyme. Previous studies have implicated fortunellin as an activator of anti-oxidant enzymes (HO-1, SOD, and CAT) through a direct action on Nrf2 and AMPK pathways, which are considered important to protect against oxidative stress [33]. In addition, fortunellin was implicated as a cardioprotective factor in diabetic animals [34]. Here, we extend the actions of this agent by reporting a direct effect of fortunellin that impairs the dimerization of 3CL-Pro SARS-CoV-2 protease and, therefore, inhibits SARS-CoV-2 replication and proliferation.

Recently, a number of 3CL-Pro mutations have been reported that alter its dimerization capacity or its enzymatic activity. However, although the amino acids that interact at the interface of the two monomers and the bonds they form have been extensively studied, the same is not true for amino acids that act allosterically at the junction of the two monomers. One such example is the amino acid Asn-28, which plays a key role in the enzymatic activity and dimerization of 3CL-Pro in SARS-CoV [35]. The Asn-28 mutation to alanine (N28A) was found to reduce the dimerization efficiency of 3CL-Pro by 19.2 times. In addition, mutations in the amino acids Leu30, Trp31, and Leu32 of 3CL-Pro to Asp, Arg, and Lys, respectively, affect the stability of the enzyme and, therefore, its functionality [36]. Another prominent mutation is that of Proline 108, as it is located in the vicinity of the proposed fortunellin-binding groove. It was reported that this mutation resulted in a 58% reduction in 3CL-Pro activity [37]. Furthermore, Asp-214-Ala and Ser-284-Ala mutations were found to allosterically affect the 3CL-Pro activity and dimerization [38].

**Figure 2:**
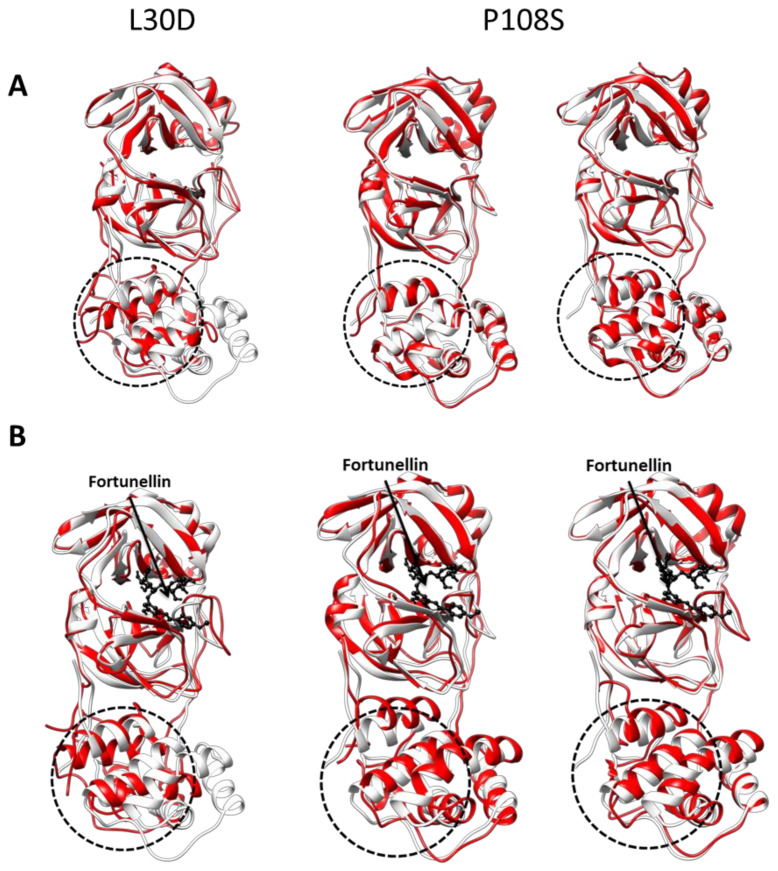
Effect of point mutations on the conformation of 3CL-Pro monomer. The crystal structure of 3CL-Pro (pdb code: 6YB7) is shown (white ribbon), while the middle structure of the mutated protein, along a 100 ns MD analysis, is shown in red. In (**A**), the structure of the unliganded protein is presented, while in (**B**), the bound structure of the protein is shown. Fortunellin is presented in ball-and-sticks while the dimerization interface of the protein is shown within a dotted circle.

Here, we have used the structure of the wild-type 3CL-Pro as an initial model (pdb: 6YB7) and performed all the above mutations, followed by fortunellin docking, on the monomer. In the case of L30D and P108S mutations, 100 ns classical equilibrium MD trajectories were produced of the 3CL-Pro monomer prior to fortunellin binding with the same parameters reported in the Supplementary Material for the production equilibrium runs. The middle conformations of the most populous clusters for the L30D (one major cluster) and P108S (two major clusters), respectively, were retrieved employing the Jarvis–Patrick clustering method for the trajectories. Molecular docking of fortunellin was performed on these three structures. The latter are two of the most important mutations (L30D and P108S) [36,37], and thus they were analyzed in depth by optimizing the mutated structures by 100 ns molecular dynamics before fortunellin binding (Table 1, Figure 2). The MD simulation induced a significant conformational change of the 3CL-Pro molecule (Figure 2A), resulting in decreased homo-dimerization affinity (Table 1), confirming the higher accuracy of the MD approach. Fortunellin binding further allosterically modifies the dimerization interface (Figure 2B), inhibiting the homo-dimerization and, subsequently, its activation.

Our data show that, in all cases, fortunellin affinity for 3CL-Pro is not dramatically modified (in all cases, ΔG oscillates between −14.4 and −12.9 kcal/mol as compared to −13.9 of the wild type) while the binding groove of fortunellin is not modified (RMSD < 1 Å, not shown). In contrast, significant changes in the dimerization affinity were found with the ΔGs oscillating between −891 and −585 kcal/mol. In all cases, fortunellin inhibited the dimerization process (Table 1).

### 3.4. In Vitro Validation of Fortunellin Action

**Figure 3:**
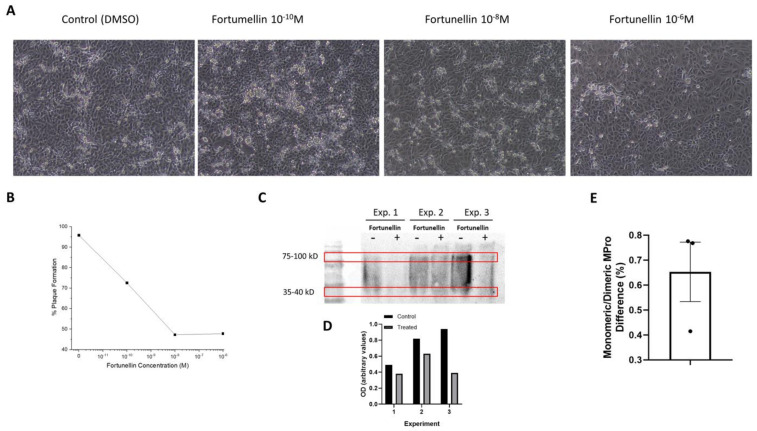
Effect of fortunellin on plaque formation of VERO cells. (**A**). SARS-CoV-2-infected VERO cells were cultured for 48 h in the absence (Control-DMSO) or the presence of different concentrations of fortunellin, as indicated. The figure presents typical microphotography for each condition repeated three times, in triplicate. The white areas in each microphotograph (acquired with an inverted phase-contrast microscope) are indicative of infected/dead cells. (**B**). Quantitation of the plaques in each condition obtained with the Fiji (ImageJ2) program. Mean ± SE of three independent assays in triplicate. (**C**). Non-denatured Western blot of 3CL-Pro in SARS-CoV-2 Vero E6-infected cells treated or not (Control) with 10^−6^ M fortunellin. Molecular markers are also presented, and the areas of the gel used for the densitometric analysis of dimerized and monomeric 3CL-Pro are denoted by red boxes. Three separate experiments are presented. (**D**). Densitometric ratio of monomeric/dimerized 3CL-Pro in non-treated and treated cells in the three experiments is shown. (**E**). Normalized differences in the monomeric/dimerized 3CL-Pro densitometric ratios between treated and untreated cells.

Fortunellin was not toxic in VERO cell cultures across the tested range (Supplemental Figure S3). The slight decrease in cell viability observed was not significant. Infection of VERO cells with SARS-CoV-2 induced significant plaque formation after 48 h. The addition of different concentrations of fortunellin resulted in a significant decrease in cell destruction, which also resulted in reduced plaque formation (Figure 3A,B). In addition, Western blot analysis of the monomeric and dimerized 3CL-Pro revealed a significant increase in the monomeric protein (a decrease in the ratio of monomeric/dimerized signal Figure 3C,D, *p* < 0.05 in Wilcoxon, one sample *t*-test, Figure 3E) in cells treated with 10^−6^ M fortunellin. These results indicate a reduced number of infected cells and the potency of fortunellin to inhibit viral proliferation with a concomitant reduced 3CL-Pro dimerization as suggested by our in silico prediction described above.

### 3.5. Beyond Fortunellin: The Structural Analogs

We have further interrogated the ZINC database for natural products with fortunellin as bait in order to identify additional molecules with 3CL-Pro binding activity. We have identified 16 additional related natural compounds for which in-silico binding on 3CL-Pro revealed a binding affinity similar to that of fortunellin (ΔG ranging from −14.2 to −11.2 kcal/mol as compared to −13.9 for fortunellin, Table S3). Analysis of their pharmacodynamic–pharmacokinetic properties (Table S4), when evaluated in the SwissADME site, show that: (1) all substances are water soluble with low absorption in the GI tract; (2) none of the compounds are toxic as they do not interact with the CYPs involved in drug metabolism; and(3) all identified compounds as well as fortunellin are substrates of the P-glycoprotein 1 (Pgp or MDR1) multidrug transporter, suggesting rapid elimination from the intracellular medium and explaining the significant, although moderate, (~50%) action of fortunellin on 3CL-Pro dimerization. Among them, apiin (ZINC3983878) and rhoifolin (ZINC3978800) have been previously studied for their biological effects.

*Apiin* (*apigenin*-*7*-*apioglucoside*) is a natural flavonoid, a diglycoside of the flavone apigenin, that is isolated from the leaves of *Apium graveolens* var. *dulce* (celery) and *Petroselinum crispum* (parsley). Apiin extracted from celery exhibited anti-inflammatory properties as apiin showed strong inhibitory activity on inducible nitric oxide synthase (iNOS) expression and nitrite (NO) production when added before LPS stimulation of J774.A1 cells [39]. In mice models, apiin had remarkable scavenging activity on maleic dialdehyde (MDA) and lipofuscin (LPF), promoted total antioxidant capacity (TAOC), and significantly enhanced the activities of superoxide dismutase (SOD), glutathione peroxidase (GSH-Px), and catalase (CAT) [40] by exerting radical scavenging activity greater than that of ascorbic acid [41] and vitamin E [40]. Apiin also showed a marked antihypertensive effect in experimental pulmonary hypertension in dogs [42] and in anti-influenza virus activity in vitro through inhibition of neuraminidase (NA) [43]. Besides, the role of apiin in cardiovascular activity as an antiarrhythmic and anti-ischemic agent has also been reported [44]. In view of our results, apiin might therefore be a strong drug candidate as it inhibits SARS-CoV-2 virus and also tackles major disease symptoms of COVID-19.

*Rhoifolin* (*apigenin 7*-*O*-*neohesperidoside*) is a neohesperidoside, a dihydroxyflavone, and a glycosyloxyflavone that was first isolated from the plant *Rhus succedanea* (a sumac or wax tree originating in Asia but also found in Australia and New Zealand) [45]. Recently, rhoifolin was found to efficiently block the enzymatic activity of SARS-CoV 3CL-Pro [46] with a methodology similar to that used in the present study. In addition, rhoifolin was reported to inhibit CVB3 infection, a primary cause of viral myocarditis in humans. In addition, it was found to reduce inflammation by significantly decreasing prostaglandin E2 and releasing pro-inflammatory cytokines (TNF-α, IL-1β, and IL-6) [47,48]. Rhoifolin isolated from *Citrus grandis* leaves was beneficial in metabolic diseases, including type II diabetes, by enhancing adiponectin secretion, tyrosine phosphorylation of insulin receptor-β, and GLUT4 translocation [49]. Rhoifolin also caused a decrease in mean aortic pressures of the arterial and pulmonary capillary pressures as well as a decrease in heart rates in dogs [50]. Moreover, a previous study has demonstrated that rhoifolin can have an inhibitory effect on angiotensin-converting enzyme (ACE) activity, which plays a key role in the regulation of arterial blood pressure [50].

Very recently, Qiao et al. [5] suggested a series of bicycloproline analogs of the approved hepatitis C antivirals boceprevir and telaprevir as 3CL-Pro inhibitors. Two compounds (MI-09 and MI-30) were selected and shown to exhibit antiviral activity in vitro and in vivo. Interestingly, both compounds have an ~80% homology with fortunellin (Supplemental Figure S2B). We have identified similar phenolic or polar benzene groups and polar atoms that form the basic skeletons. However, the atomic volume of fortunellin is higher as compared to both MI-9 and MI-30 compounds. We have further found that fortunellin binds to the active site of 3CL-Pro, in which compounds PI-09 and PI-30 also bind (Supplemental Figure S2A), albeit with a lower affinity for fortunellin (−11.8 as compared to −13.9 kcal/mol). Alternatively, we have also probed the binding of compounds MI-09 and MI-30 at the fortunellin-binding site of 3CL-Pro. MI-09 and MI-30 bind to the fortunellin-binding pocket with a higher affinity than on the active site of 3CL-Pro (ΔG, −16.190 and −14.6 Kcal/mol, respectively, Table S5, Figure S2C–F), providing an alternative model for the mode of the action of these inhibitors against COVID-19. In addition, a dimerization simulation revealed that the compounds MI-09 and MI-30 lead to the inhibition of dimerization of 3CL-Pro.

## 4. Conclusions

In this contribution, we have explored the conformational space of the SARS-CoV-2 main protease (3CL-Pro) in relation to its dimerization. We provide the complete set of collective variables that can serve as a basis to explore key conformations of the homodimer. Subsequent in vitro tests confirmed our in silico strategy and identified a series of natural flavonoids—polyphenols (fortunellin, apiin, rhoifolin)—which, in the form of drugs or dietary supplements, may target the 3CL-Pro dimerization and act as an effective strategy against the devastating SARS-CoV-2 infections in humans.

## Figures and Tables

**Table 1:** Effects of reported point mutations of 3CL-Pro on fortunellin binding and the homo-dimerization capacity of 3CL-Pro monomers.

Mutation	ΔG ^1,2^	Dimerization ΔG ^2^	Dimerization	References
Fortunellin		Absent	Present	
Wild Type	−13.936	−683.89	X	
N28A	−13.694	−733.26	X	[35]
L30D	−13.381	−787.41	X	[36]
W31R	−12.969	−845.34	X	[36]
L32K	−14.180	−585.04	X	[36]
P108S	−13.142	−891.67	X	[37]
N214A	−14.461	−845.14	X	[38]
S284A	−14.753	−760.32	X	[38]
molecular dynamics middle structures				
L30D	−12.682	−683.03	X	
P108S_Model A	−13.902	−564.94	X	
P108S_Model B	−13.572	−685.19	X	

^1^ ΔG of fortunellin binding in a fully flexible model; ^2^ Data are expressed in kcal/mol.

## Data Availability

All data and analyses are available within the manuscript and the supporting information, or upon reasonable request to the corresponding authors.

## Supplementary Materials

The following are available online. List S1: PDB codes for 3CL-Pro crystals, Table S2: global and local RMSD values, Table S3: ZINC compounds of interest, Table S4: ADME characteristics of 16 compounds, Table S5: energetics of binding of fortunellin and analogs on 3CL-Pro, Figure S1: Superposition of 10 different 3CL-PRO models, Figure S2: The binding pocket of Fortunellin, Figure S3: VERO cell viability.

## Sample Availability

Samples of the compounds are not available from the authors.
