# Supplementary material for: Natural Polyphenols Inhibit the Dimerization of the SARS-CoV-2 Main Protease: The Case of Fortunellin and Its Structural Analogs
Source: Molecules. 2021 Oct 7;26(19):6068. doi: 10.3390/molecules26196068 (PMC8512273; doi:10.3390/molecules26196068)
Supplement: Supplementary file 1 [file molecules-26-06068-s001.zip › molecules-1356669-supplementary.pdf]

Supplemental Material

Natural polyphenols inhibit the dimerization of the SARS-CoV-2 main protease: the case of fortunellin and its structural analogs

Athanasios A. Panagiotopoulos[1], Ioannis Karakasiliotis[2], Danai-Maria Kotzampasi[1], Marios Dimitriou[2], George Sourvinos[3,4], Marilena Kampa[1,4], Stergios Pirintsos[4,5,6], Elias Castanas[1,4,*] and Vangelis Daskalakis[7,*]

[1] Laboratory of Experimental Endocrinology, University of Crete, School of Medicine, Heraklion, Greece

[2] Laboratory of Biology, School of Medicine, Democritus University of Thrace, Alexandroupolis, Greece

[3] Laboratory of Virology, University of Crete, School of Medicine, Heraklion, Greece

[4] Nature Crete Pharmaceuticals, Heraklion, Greece

[5] Department of Biology, University of Crete, 71409 Heraklion, Greece

[6] Botanical Garden, University of Crete, Rethymnon, Greece

[7] Department of Chemical Engineering, Cyprus University of Technology, Limassol, Cyprus

* Correspondence: castanas@uoc.gr; evangelos.daskalakis@cut.ac.cy

Contents

Methods 3
  Molecular Docking 3
    SARS-CoV-2 3CL-Pro main protease and Ligand Preparation 3
    3CL-Pro/ Ligand docking 3
  Molecular Dynamics computational protocol 3
    Model setup 3
    Equilibration-Production Molecular Dynamics setup 4
    Markov State Model Analysis 5
    Enhanced Molecular Dynamics Sampling 9
Supplemental list S1 9
Supplemental Table S2 10
Supplemental Table S3 12
Supplemental Table S4 17
Supplemental Table S5 19
Supplemental Figure S1 20
Supplemental Figure S2 21
Supplemental Figure S3 23
References 24

Methods

Molecular Docking

SARS-CoV-2 3CL-Pro main protease and Ligand Preparation

The sequence of SARS-CoV-2 main protease 3CL-Pro, in fasta format, was retrieved from the NCBI protein database (https://www.ncbi.nlm.nih.gov/protein/) and introduced to the Swiss Model Biospace (http://swissmodel.expasy.org/interactive).[1] The main SARS-CoV-2 protease 3CL-Pro has many available crystal structures and the system, presented in Supplemental Table 1. Codes correspond to data stored in the Protein Data Bank (https://www.rcsb.org/).[2] In this work, we have used the 6YB7 unliganded crystal analyzed with X-ray diffraction for the initial docking studies.

Ligands were retrieved from the ZINC database (http://zinc.docking.org/),[3] in a canonical smiles format. Novel molecules were designed in ChemBioDraw (v12.0, Perkin Elmer, Boston, MA, free for Academic use from the University of Cambridge). Then, pdb or mol2 files of the ligands were created with the Open Babel program (http://openbabel.org).[4]

3CL-Pro/ Ligand docking

SARS-CoV-2 main protease and ligand(s) were uploaded to the GalaxyWebserver (http://galaxy.seoklab.org/), and a fully flexible docking (involving the receptor and the ligand) was performed. [5–10] The server uses an algorithm based on the GalaxyDock2 docking [11], which, after an automatic prediction of the ligand-binding pocket, permits a full ligand/receptor flexibility during binding simulation. This step is followed by optimization and subsequent refinement through a specific algorithm named GalaxyRefine [6,12], which permits a protein-ligand structure refinement by applying iterative side chain repacking and overall structure relaxation.[6,13] The best solution (usually denoted as “pose 1”) was retained and further used.

Molecular Dynamics computational protocol

Model setup

The crystal structures of SARS-CoV-2 main protease 3CL-PRO (pdb codes 6LU7[14] and 6YB7) were used as initial coordinates to build the models. The pdb structures refer to one monomer without and with inhibitor, respectively. For consistency, the inhibitor was removed from the crystal structure of 3CL-Pro (6LU7). [14] Our choice of coordinates was based on the completeness of the resolved 3CL-Pro sequence and the quality of chains (at least 90%). To build the 3CL-Pro homodimer, we structurally aligned either two 6lu7, two 6yb7 monomers, on a reference dimer. [15] The protonation states of titratable residues were simulated at neutral pH; thus, all Glu and Asp residues were left deprotonated, except Glu-290, which was protonated in accordance with the PDB2PQR (propka 3.0 method, pH 7.3) predictions. [16] His-41, His-163, His-172, and His-246 were protonated only at the Nε site. The rest of His residues were protonated only at the Nδ sites, to maintain the hydrogen bonding network

within the crystal structures. All crystallographic water molecules are retained within each crystal structure. Four samples were, thus prepared, in a consistent way (two monomers, two homodimers). The all-atom models, as defined previously, were embedded in triclinic boxes of around 7.2nm x 11.2nm x 8.0nm (monomer) or 12.3nm x 12.3 nm x 12.3 nm (dimer) in the x, y, and z dimensions, respectively. Up to around 57000 TIP3P water molecules [17] were used to hydrate each protein. Ion (K^+, Cl^-) concentration was set at the value of 150 mM to mimic the physiological salt content for the monomer or homodimer models, in addition to zero, or excess concentrations of KCl, NaCl, and $CaCl_2$ at 0, 150, 300, 400, and 500 mM only for the monomer models. The various anionic strengths were only employed to indirectly 'enhance' the sampled conformational space of the 3CL-Pro. A surplus of K^+ was also added to neutralize the protein charges in each sample, resulting in simulation unit boxes of 62400 (monomer) or 181800 (dimer) atoms. The Amber03 [18] protein force field was used for the residues and ions. The Amber03 parameters for the natural products were derived based on the ACPYPE algorithm. [19]

Equilibration-Production Molecular Dynamics setup

The equilibration-relaxation for the all-atom systems is employed based on a published protocol for water-soluble proteins.[20] This contains a steepest descend energy minimization with a tolerance of 0.5 kJ mol^{-1} for 1000 steps, and a sequence of isothermal (nVT), isothermal-isobaric (nPT) runs with the gradual relaxation of the constraints on protein-heavy atoms (from 10^4 in steps 1–2 to 10^3 kJ mol^{-1} nm^{-2} in step 4) and Cα atoms (from 10^3 in step 5 to 10^2 in step 6, 10 in step 7, 1 in step 8, and 0 kJ mol^{-1} nm^{-2} in step 9) for around 30 ns, with a time step of 1.0 fs (steps 1–4) and 2.0 fs (steps 5–9). In detail: (**step 1**) Constant density and temperature (nVT) Brownian dynamics (BD) at 100 K for 50 ps that employs the Berendsen thermostat, [21] with a temperature coupling constant at 1.0 fs. (**steps 2–3**) Two short constant density (nVT) and constant pressure (nPT) runs for 100 ps each, with a weak coupling Berendsen thermostat and barostat [21] at 100 K employing time coupling constants of 0.1 ps for the temperature and isotropic 50.0 ps coupling for the pressure with a compressibility of $4.6x10^{-5}$. (**step 4**) Heating from 100 to 250 K in a constant density ensemble (nVT) for 3 ns employing the v-rescale thermostat, [22] with a time coupling constant of 0.1 ps. (**step 5**) Heating from 250 to 310K in a constant pressure ensemble (nPT) for 2 ns, employing the v-rescale thermostat [22] and Berendsen barostat,[21] with time coupling constants of 0.1 ps for the temperature and 2.0 ps for the pressure, removing also all but the Cα-atom protein position restraints. (**step 6**) Equilibration at 310K (0.1 ps temperature coupling constant) for 5 ns (nPT, 1 atm, 2.0 ps coupling constant for pressure. (**steps 7–8**) Equilibration at 310K (0.5 ps temperature coupling constant) for 5 ns (nPT, 1 atm, 2.0 ps coupling constant for pressure). (**step 9**) Equilibration at 310K (0.5 ps temperature coupling constant) for 10 ns (nPT, 1 atm, 2.0 ps coupling constant for pressure). The barostats and thermostats employed for steps 6–9 were the same as in the production trajectories that follow.

For the production of all-atom classical Molecular Dynamics (MD), Newton's equations of motion are integrated with a time step of 2.0 fs at 310K. All production simulations are run with the leap-frog integrator in GROMACS 2020[23] for 3.0 µs each. They were performed at the constant pressure nPT ensemble, with isotropic coupling (compressibility at $4.5x10^{-5}$) employing the v-rescale thermostat[22] (310K, temperature coupling constant 0.5) and the *Parrinello–Rahman* barostat [24] (1 atm, pressure coupling constant 2.0). Details for parameters can be found in earlier work. [20] The first 500 ns were considered further equilibration from each independent trajectory per sample and were disregarded in the analysis, based also on the RMSD fluctuations (a plateau is reached roughly beyond 100-200ns depending on the trajectory). Van der Waals interactions were smoothly switched to zero between 1.0-1.2 nm with the VERLET cut-off scheme. Electrostatic interactions were truncated at 1.2 nm (short-range), and long-range contributions were computed within the PME approximation. [25,26] Hydrogen bond lengths were constrained employing the LINCS algorithm. [27]

Markov State Model Analysis

We obtained a series of MD equilibrium trajectories of the 3CL-Pro monomers (20 x 3.0µs = 60µs) of SARS-CoV-2 main protease under different salts/ ionic strengths, without inhibitors. These should have explored a major part of the 3CL-Pro conformational phase space. We combined the all-atom MD simulations with Markov state model (MSM) theory[28–30] in order to enable the extraction of long-time-scale individual monomer dynamics from rather short-time-scale MD trajectories of different states. The application and accuracy of the powerful MSM theory have been presented in many cases also by experiments that include protein–protein or protein–drug binding kinetics, as well as protein folding rates and protein dynamics. [31–34] Our objective was to approximate the slow dynamics in a statistically efficient manner. Thus, a lower-dimensional representation of our simulation data was necessary. In order to reduce the dimensionality of our feature space, we employed the time-structure independent components analysis (tICA), which yields a representation of our molecular simulation data with reduced dimensionality and can greatly facilitate the decomposition of our system into the discrete Markovian states necessary for the MSM estimation. The conformations of the system were projected on these slowest modes as defined by the tICA method, then the trajectory frames were clustered into 100 cluster-centers (microstates) by k-means clustering, as implemented in PyEMMA. [35] Conformational changes of a system can be simulated as a Markov chain if the transitions between the different conformations are sampled at long enough time intervals so that each transition is Markovian. This means that a transition from one conformation to another is independent of the previous transitions. Therefore, an MSM is a memoryless model. The uncertainty bounds were computed using a Bayesian scheme.[36,37] We found that the slowest implied timescales converge quickly and are constant within a 95% confidence interval for lag times above 50ns. The validation procedure is a standard approach in the MSM

field, a lag time of 50 ns was selected for Bayesian model construction, and the resulting models were validated by the Chapman–Kolmogorov (CK) test. Subsequently, the resulting MSMs were further coarse grained into a smaller number of three metastable states or microstates using PCCA++ as implemented in PyEMMA. [35] The optimum number of microstates (three) was proposed based on the VAMP2-score. [38] Both the convergence of the implied timescales as well as the CK test confirm the validity and convergence of the MSM. The CK test indicates that predictions from the built MSM agree well with MSMs estimated with longer lag times. Thus, the model can describe well the long-time-scale behavior of our system within error. The tICA method identified the torsional angles of the following 3CL-Pro residues: 3, 4, 5, 6, 84, 135, 141, 164, 167, 171, 175, 178, 179, 180, 190, 195, 217, 284, 285, 286, 290, 291, 300, and 301 as the most important features, by setting a series of thresholds for the coefficients in the tICA vectors. At first, we set a threshold of 0.09. We continued by setting a threshold of 0.04 for the coefficients in the tICA vectors of the filtered data and afterwards a threshold of 0.085. Finally, we set a threshold of 0.075, and thus, we concluded in the previously referred 3CL-Pro residues. For the selection of these thresholds, we checked for different thresholds, the VAMP2-score, and the states projected onto the first two independent components. We report the exact functions of the first two tICA components as a linear combination of cosine/ sine functions of the associated torsionals:

CV1 = (0.22245479085963174)*COS(PHE3PHI)+(-
0.16733307515910162)*SIN(PHE3PHI)+(0.1432275835003262)*COS(PHE3PSI)+(0.200886899869637
5)*SIN(PHE3PSI)+(0.19936685751147043)*COS(ARG4PHI)+(-
0.09327647115497929)*SIN(ARG4PHI)+(-0.0930358300031665)*COS(ARG4PSI)+(-
0.08538905470780045)*SIN(ARG4PSI)+(0.10166329580502668)*COS(LYS5PHI)+(-
0.09568173783678022)*SIN(LYS5PHI)+(-0.09287774945984135)*COS(LYS5PSI)+(-
0.15790698786616095)*SIN(LYS5PSI)+(-
0.0956189898931311)*COS(MET6PHI)+(0.025245999648597445)*SIN(MET6PHI)+(0.0408771827310
96175)*COS(MET6PSI)+(-
0.0006973055782739627)*SIN(MET6PSI)+(0.12585421365826116)*COS(ASN84PHI)+(0.1980318831
213358)*SIN(ASN84PHI)+(0.08785335907805712)*COS(ASN84PSI)+(-
0.1678608618927701)*SIN(ASN84PSI)+(0.3615169358035203)*COS(THR135PHI)+(0.2355840179344
413)*SIN(THR135PHI)+(0.06937744998780684)*COS(THR135PSI)+(-
0.08624991337144639)*SIN(THR135PSI)+(0.06993246492427758)*COS(LEU141PHI)+(-
0.023980684220480688)*SIN(LEU141PHI)+(-0.0028980923592885344)*COS(LEU141PSI)+(-
0.11124570694911744)*SIN(LEU141PSI)+(0.12367941924553509)*COS(HIS164PHI)+(0.1100289626
0080477)*SIN(HIS164PHI)+(0.1736049601096124)*COS(HIS164PSI)+(-
0.10071229902364524)*SIN(HIS164PSI)+(0.04589771207808335)*COS(LEU167PHI)+(0.0319038117
3082644)*SIN(LEU167PHI)+(0.010408062573537607)*COS(LEU167PSI)+(-
0.001452825004362783)*SIN(LEU167PSI)+(-
0.035449253177574046)*COS(VAL171PHI)+(0.0397831323983392)*SIN(VAL171PHI)+(-
0.01487903934140085)*COS(VAL171PSI)+(-
0.034102380825128384)*SIN(VAL171PSI)+(0.030847079909921624)*COS(THR175PHI)+(-
0.020040648643118112)*SIN(THR175PHI)+(0.12177024626152966)*COS(THR175PSI)+(-
0.13229839249150296)*SIN(THR175PSI)+(0.11507939509465313)*COS(GLU178PHI)+(0.1013714392

35159)*SIN(GLU178PHI)+(-
0.09137875539957069)*COS(GLU178PSI)+(0.11214339094322172)*SIN(GLU178PSI)+(-
0.09294920607798879)*COS(GLY179PHI)+(0.28997810094722837)*SIN(GLY179PHI)+(-
0.27573006050671983)*COS(GLY179PSI)+(-
0.09673320535071989)*SIN(GLY179PSI)+(0.010926172367439396)*COS(ASN180PHI)+(-
0.24668920154301732)*SIN(ASN180PHI)+(-
0.14912645633298552)*COS(ASN180PSI)+(0.3816102785675176)*SIN(ASN180PSI)+(-
0.10964069317986454)*COS(THR190PHI)+(-0.009927774218497826)*SIN(THR190PHI)+(-
0.16097973924205633)*COS(THR190PSI)+(-
0.16639818413751062)*SIN(THR190PSI)+(0.08017482811083185)*COS(GLY195PHI)+(-
0.14979887114706295)*SIN(GLY195PHI)+(-0.050958292897186695)*COS(GLY195PSI)+(-
0.044354361874655926)*SIN(GLY195PSI)+(0.09987054135337944)*COS(ARG217PHI)+(-
0.12648381947691048)*SIN(ARG217PHI)+(0.08158948319042672)*COS(ARG217PSI)+(-
0.00021426690460247595)*SIN(ARG217PSI)+(0.21642336166830292)*COS(SER284PHI)+(-
0.30155833144467264)*SIN(SER284PHI)+(0.8266406109913902)*COS(SER284PSI)+(0.88748800878
14281)*SIN(SER284PSI)+(0.6649555215066218)*COS(ALA285PHI)+(0.44940445619426095)*SIN(ALA
285PHI)+(-0.7837894441282054)*COS(ALA285PSI)+(-0.7618813787744232)*SIN(ALA285PSI)+(-
0.41060021549010234)*COS(LEU286PHI)+(-
0.10014769031761044)*SIN(LEU286PHI)+(0.19732367941536325)*COS(LEU286PSI)+(0.2864165371
4402343)*SIN(LEU286PSI)+(0.2011752565537491)*COS(GLU290PHI)+(0.08698680780975648)*SIN(
GLU290PHI)+(-
0.00570069571470415)*COS(GLU290PSI)+(0.06637101699247436)*SIN(GLU290PSI)+(0.2298963479
5972827)*COS(PHE291PHI)+(-0.17343642924439104)*SIN(PHE291PHI)+(-
0.11571250082711405)*COS(PHE291PSI)+(0.02324511232530362)*SIN(PHE291PSI)+(-
0.047478679669659275)*COS(CYS300PHI)+(0.05578968284901458)*SIN(CYS300PHI)+(-
0.1683363737052317)*COS(CYS300PSI)+(-0.2973909304454135)*SIN(CYS300PSI)+(-
0.1038665536799251)*COS(SER301PHI)+(-0.019617745122528882)*SIN(SER301PHI)+(-
0.06934626053471138)*COS(SER301PSI)+(-0.10587162831562456)*SIN(SER301PSI)

CV2 = (-0.2884241825864551)*COS(PHE3PHI)+(0.1818110423100234)*SIN(PHE3PHI)+(-
0.4677450443427827)*COS(PHE3PSI)+(-0.5483897439796918)*SIN(PHE3PSI)+(-
0.3553107265200588)*COS(ARG4PHI)+(-0.22394962064864046)*SIN(ARG4PHI)+(-
0.270179755725878)*COS(ARG4PSI)+(-0.3829402238055943)*SIN(ARG4PSI)+(-
0.2042142888426065)*COS(LYS5PHI)+(-
0.1669302788926349)*SIN(LYS5PHI)+(0.141973243784881)*COS(LYS5PSI)+(0.1421901057216299)*
SIN(LYS5PSI)+(0.0846185601658726)*COS(MET6PHI)+(0.20450776328031908)*SIN(MET6PHI)+(-
0.03312223507339774)*COS(MET6PSI)+(-0.08043115352475075)*SIN(MET6PSI)+(-
0.027921418396744165)*COS(ASN84PHI)+(-0.08339140826693826)*SIN(ASN84PHI)+(-
0.38318457142235807)*COS(ASN84PSI)+(-0.02014396174778721)*SIN(ASN84PSI)+(-
0.261602759573732)*COS(THR135PHI)+(-0.22169542813755444)*SIN(THR135PHI)+(-
0.087566095559111)*COS(THR135PSI)+(0.11762076691089288)*SIN(THR135PSI)+(-
0.3469970284275915)*COS(LEU141PHI)+(0.34989926232122653)*SIN(LEU141PHI)+(-
0.3353014777111922)*COS(LEU141PSI)+(-0.1099813590134305)*SIN(LEU141PSI)+(-
0.030401089904733867)*COS(HIS164PHI)+(-0.12811671967132546)*SIN(HIS164PHI)+(-

0.13436881803853637)*COS(HIS164PSI)+(0.17139820505530204)*SIN(HIS164PSI)+(-0.12200117274899505)*COS(LEU167PHI)+(-0.06149505441676782)*SIN(LEU167PHI)+(-0.06260851492073659)*COS(LEU167PSI)+(0.06084287820758976)*SIN(LEU167PSI)+(-0.006861594758294066)*COS(VAL171PHI)+(0.012827540881062702)*SIN(VAL171PHI)+(0.012464087205759892)*COS(VAL171PSI)+(-0.02648078463371077)*SIN(VAL171PSI)+(0.13636122184567023)*COS(THR175PHI)+(-0.16662872008089358)*SIN(THR175PHI)+(0.005026593046059509)*COS(THR175PSI)+(-0.020823512651369957)*SIN(THR175PSI)+(-0.15569457005305837)*COS(GLU178PHI)+(-0.12910762053786512)*SIN(GLU178PHI)+(0.195962425618426)*COS(GLU178PSI)+(-0.1674075865238855)*SIN(GLU178PSI)+(-0.05538839797914528)*COS(GLY179PHI)+(-0.2984068151828335)*SIN(GLY179PHI)+(0.4196490331550077)*COS(GLY179PSI)+(0.2097945401494899)*SIN(GLY179PSI)+(-0.021574332218825033)*COS(ASN180PHI)+(0.3273623222055997)*SIN(ASN180PHI)+(0.08717870414956289)*COS(ASN180PSI)+(-0.47359288885540496)*SIN(ASN180PSI)+(-0.04538558390983336)*COS(THR190PHI)+(-0.008683270082020167)*SIN(THR190PHI)+(0.271129910670838)*COS(THR190PSI)+(0.27549665651127636)*SIN(THR190PSI)+(0.0780842690641956)*COS(GLY195PHI)+(0.044233409296374225)*SIN(GLY195PHI)+(0.13353598315320972)*COS(GLY195PSI)+(0.04465259963400809)*SIN(GLY195PSI)+(0.027701779053662518)*COS(ARG217PHI)+(0.03397617632773808)*SIN(ARG217PHI)+(-0.055406779213062196)*COS(ARG217PSI)+(-0.15249136969809435)*SIN(ARG217PSI)+(0.15917442405868654)*COS(SER284PHI)+(0.07317395309230672)*SIN(SER284PHI)+(0.050094307429749026)*COS(SER284PSI)+(0.19617816860701598)*SIN(SER284PSI)+(0.1826732916695155)*COS(ALA285PHI)+(-0.035338601991516574)*SIN(ALA285PHI)+(-0.19924762948366187)*COS(ALA285PSI)+(-0.1564466277523662)*SIN(ALA285PSI)+(-0.0443686239963879)*COS(LEU286PHI)+(-0.10931048704118541)*SIN(LEU286PHI)+(0.45176938273388506)*COS(LEU286PSI)+(0.4751207479763982)*SIN(LEU286PSI)+(0.1954750353189944)*COS(GLU290PHI)+(0.28698450313441914)*SIN(GLU290PHI)+(0.1178561297010931)*COS(GLU290PSI)+(-0.11293348016651641)*SIN(GLU290PSI)+(-0.23109368829696822)*COS(PHE291PHI)+(0.2905179445957612)*SIN(PHE291PHI)+(0.2414087396867373)*COS(PHE291PSI)+(-0.19323071296694158)*SIN(PHE291PSI)+(-0.06723387446469688)*COS(CYS300PHI)+(0.08472356860436044)*SIN(CYS300PHI)+(0.11286902793988102)*COS(CYS300PSI)+(0.33756361790231243)*SIN(CYS300PSI)+(0.07472554678618155)*COS(SER301PHI)+(-0.04927948273422832)*SIN(SER301PHI)+(0.13667888455911714)*COS(SER301PSI)+(0.1947392228687667)*SIN(SER301PSI)

Based on the 3CL-Pro + fortunellin classical molecular dynamics simulations, the residues that seem to affect the 3CL-Pro + fortunellin dynamics are derived based on the same MSM/ tICA protocol, as described above in the absence of the inhibitor. These include: 44, 48, 53, 82, 83, 84, 111, 112, 118, 137, 138, 139, 141, 159, 182, 238, 239, 240, 286, 287, 288, 289, and 291. At first, we set a threshold of 0.075. We continued by setting a threshold of 0.12 for the coefficients in the tICA vectors of the filtered data, a threshold of 0.18, a threshold of 0.065, and afterwards a threshold of 0.135. Finally, we set a threshold of 0.125, and thus, we concluded in the previously referred residues. However, the 3CL-Pro states in the presence of fortunellin, reported in Figure 1E of the main manuscript, are derived based on the projection

of the 3CL-Pro + fortunellin trajectory data on the aforementioned phase space of the CV1, CV2 functions.

Enhanced Molecular Dynamics Sampling

To enhance the conformational sampling on the 3CL-Pro homodimer, we employed the parallel tempering metadynamics in the well-tempered ensemble (PTmetaD-WTE) method.[39–42] Nine replicas per sample were run at 310, 320, 330, 341, 352, and 363K, 375K, 387K and 400K in which only the potential energy (PE) was initially biased (bias factor 120) to achieving large fluctuations in PE and replica overlaps. Replicas were allowed to exchange every 1000 steps for 0.2μs each, which gave an exchange probability of around 20% in the WTE. The obtained bias was saved and used for the subsequent PTmetaD production runs for another 0.5μs per sample/ replica. Nine replicas were again considered at the same temperatures. Including the equilibration time at reach replica, a cumulative simulation time of 10μs was achieved. An exchange was attempted every 1000 steps, which gave an exchange probability between replicas at around 20%, consistent with the large sample sizes. The Collective Variables (CVs) chosen for the PTmetaD runs were the first two tICA vectors presented above (CV1/ CV2). A combination of the GROMACS 2020/ PLUMED 2.5[43] engines was employed. A bias factor of 25 at the well-tempered ensemble, along with Gaussians of 1.2 kJ/mol initial height, and sigma values (width) of 0.25 in the CV space, deposited every 2 ps, was employed. The grid space for both CVs is defined between -4 and 4 at a resolution of 0.05. Four different PTmetaD-WTE runs were performed for the pdb 6yb7/ 6lub7-based dimers at 150mM KCl without inhibitor.

Supplemental list S1

PDB codes of 3CL-PRO crystals, deposited in the PDB databank, accessed at July 3, 2020.

5r7y, 5r7z, 5r80, 5r81, 5r82, 5r83, 5r84, 5r8t, 5re4, 5re5, 5re6, 5re7, 5re8, 5re9, 5rea, 5reb, 5rec, 5red, 5ree, 5ref, 5reg, 5reh, 5rei, 5rej, 5rek, 5rel, 5rem, 5ren, 5reo, 5rep, 5rer, 5res, 5ret, 5reu, 5rev, 5rew, 5rex, 5rey, 5rez, 5rf0, 5rf1, 5rf2, 5rf3, 5rf4, 5rf5, 5rf6, 5rf7, 5rf8, 5rf9, 5rfa, 5rfb, 5rfc, 5rfd, 5rfe, 5rff, 5rfg, 5rfh, 5rfi, 5rfj, 5rfk, 5rfl, 5rfm, 5rfn, 5rfo, 5rfp, 5rfq, 5rfr, 5rfs, 5rft, 5rfu, 5rfv, 5rfw, 5rfx, 5rfy, 5rfz, 5rg0, 5rg1, 5rg2, 5rg3, 5rgg, 5rgh, 5rgi, 5rgj, 5rgk, 5rgl, 5rgm, 5rgn, 5rgo, 5rgp, 5rgq, 5rgr, 5rgs, 5rgt, 5rgu, 5rgv, 5rgw, 5rgx, 5rgy, 5rgz, 5rh0, 5rh1, 5rh2, 5rh3, 5rh4, 5rh5, 5rh6, 5rh7, 5rh8, 5rh9, 5rha, 5rhb, 5rhc, 5rhd, 5rhe, 5rhf, 6lu7, 6lze, 6m03, 6m0k, 6m2n, 6m2q, 6w63, 6wnp, 6wqf, 6wtj, 6wtk, 6wtm, 6wtt, 6xa4, 6xb0, 6xb1, 6xb2, 6xbg, 6xbh, 6xbi, 6xch, 6y2e, 6y2f, 6y2g, 6y84, 6yb7, 6ynq, 6yt8, 6yvf, 6yz6, 6z2e, 7bqy, 7bro, 7brp, 7brr, 7buy, 7c8r, 7c8t

Supplemental Table S2

Table S2 presents the global and local RMSD values at the dimerization domain) of 3CL-Pro monomer at different time-frames, as determined by molecular dynamics. The interaction of fortunellin in each pose is also shown as changes of the Gibbs free energy changes (ΔG).

Model	Time (µs)	RMSD Total	RMSD Local	ΔG (kcal/mol)
0	0	0.000	0.000	-13.936
1	3	1.637	1.235	-11.199
2	6	2.248	3.290	-12.144
3	9	1.954	1.515	-10.704
4	12	1.421	1.292	-14.213
5	15	1.756	1.085	-11.611
6	18	1.706	0.998	-11.987
7	21	2.292	2.608	-14.658
8	24	2.694	2.757	-13.699
9	27	1.845	1.230	-13.563
10	30	1.821	1.268	-11.468
11	33	2.031	1.344	-12.760
12	36	1.924	1.604	-12.518
13	39	1.888	1.269	-11.579
14	42	2.544	2.686	-12.165
15	45	1.897	1.463	-11.507
16	48	2.122	1.883	-12.423
17	51	1.965	1.420	-11.478

18	54	1.924	1.534	-11.997
19	57	1.915	1.263	-12.070
20	60	1.874	1.385	-12.660

Supplemental Table S3

ZINC number, formula, SMILES and binding affinity for 3CL-Pro (as expressed by Gibb's free energy, ΔG, change in kcal/mol) of the sixteen (16) compounds from the ZINC database for natural products.

Compound	2D Structure	Smiles	ΔG (Kcal/mol)
ZINC4349204 (Fortunellin)	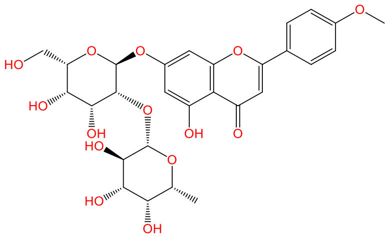	COc1ccc(-c2cc(=O)c3c(O)cc(O[C@@H]4O[C@@H](CO)[C@@H](O)[C@@H](O)[C@H]4O[C@@H]4O[C@H](C)[C@H](O)[C@H](O)[C@H]4O)cc3o2)cc1	-13.936
ZINC4349029	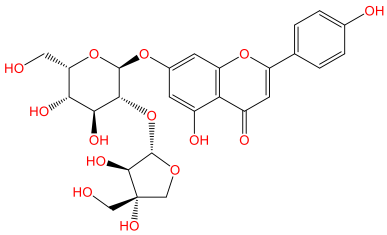	O=c1cc(-c2ccc(O)cc2)oc2cc(O[C@@H]3O[C@@H](CO)[C@@H](O)[C@H](O)[C@H]3O[C@@H]3OC[C@@](O)(CO)[C@H]3O)cc(O)c12	-14.241
ZINC4349031	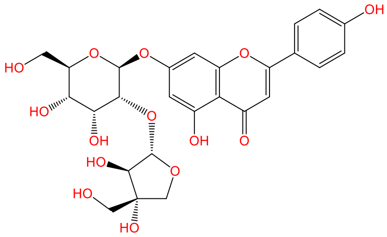	O=c1cc(-c2ccc(O)cc2)oc2cc(O[C@@H]3O[C@H](CO)[C@@H](O)[C@@H](O)[C@H]3O[C@@H]3OC[C@@](O)(CO)[C@H]3O)cc(O)c12	-12.352

ZINC4349034	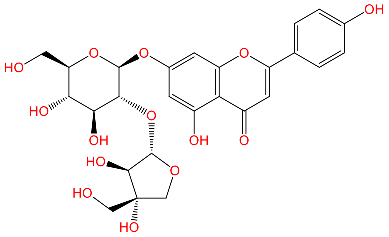	O=c1cc(-c2ccc(O)cc2)oc2cc(O[C@@H]3O[C@H](CO)[C@@H](O)[C@H](O)[C@H]3O[C@@H]3OC[C@@](O)(CO)[C@H]3O)cc(O)c12	-12.451
ZINC3983878 (Apiin)	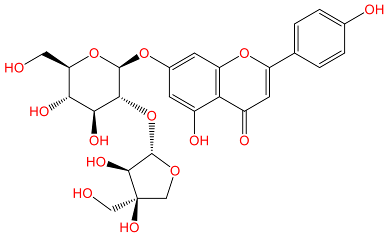	O=c1cc(-c2ccc(O)cc2)oc2cc(O[C@@H]3O[C@H](CO)[C@@H](O)[C@H](O)[C@H]3O[C@@H]3OC[C@](O)(CO)[C@H]3O)cc(O)c12	-12.604
ZINC4349207	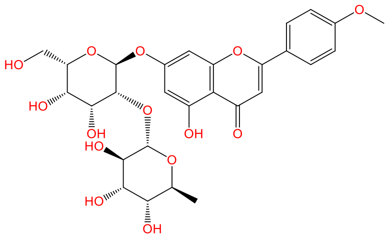	COc1ccc(-c2cc(=O)c3c(O)cc(O[C@@H]4O[C@@H](CO)[C@@H](O)[C@@H](O)[C@H]4O[C@@H]4O[C@@H](C)[C@H](O)[C@H](O)[C@H]4O)cc3o2)cc1	-11.539
ZINC4349211	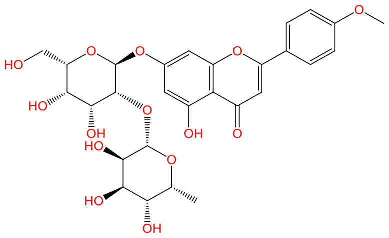	COc1ccc(-c2cc(=O)c3c(O)cc(O[C@@H]4O[C@@H](CO)[C@@H](O)[C@@H](O)[C@H]4O[C@@H]4O[C@H](C)[C@H](O)[C@@H](O)[C@H]4O)cc3o2)cc1	-11.545

ZINC4349214	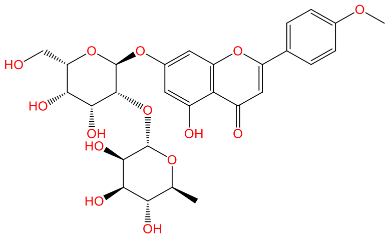	COc1ccc(-c2cc(=O)c3c(O)cc(O[C@@H]4O[C@@H](CO)[C@@H](O)[C@@H](O)[C@H]4O[C@@H]4O[C@@H](C)[C@H](O)[C@@H](O)[C@H]4O)cc3o2)cc1	-12.150
ZINC4349623	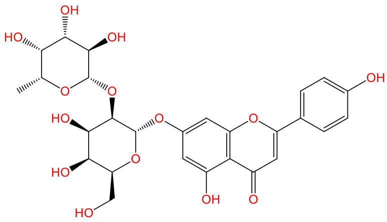	C[C@H]1O[C@@H](O[C@H]2[C@H](Oc3cc(O)c4c(=O)cc(-c5ccc(O)cc5)oc4c3)O[C@@H](CO)[C@@H](O)[C@H]2O)[C@H](O)[C@@H](O)[C@H]1O	-12.970
ZINC4349627	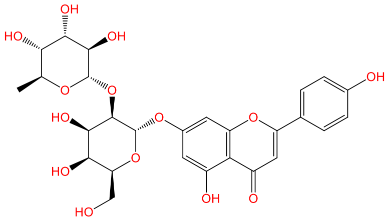	C[C@@H]1O[C@@H](O[C@H]2[C@H](Oc3cc(O)c4c(=O)cc(-c5ccc(O)cc5)oc4c3)O[C@@H](CO)[C@@H](O)[C@H]2O)[C@H](O)[C@@H](O)[C@H]1O	-13.200
ZINC4349630	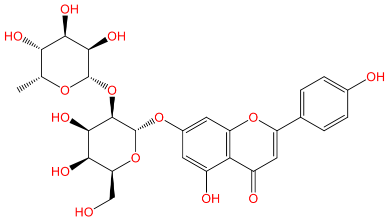	C[C@H]1O[C@@H](O[C@H]2[C@H](Oc3cc(O)c4c(=O)cc(-c5ccc(O)cc5)oc4c3)O[C@@H](CO)[C@@H]	-12.229

		(O)[C@H]2O)[C@H](O)[C@H](O)[C@H]1O	
ZINC4349633	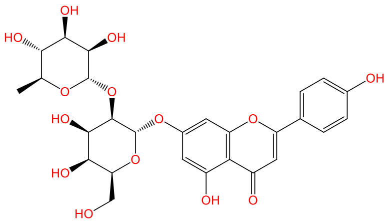	C[C@@H]1O[C@@H](O[C@H]2[C@H](Oc3cc(O)c4c(=O)cc(-c5ccc(O)cc5)oc4c3)O[C@@H](CO)[C@@H](O)[C@H]2O)[C@H](O)[C@H](O)[C@H]1O	-12.937
ZINC4534057	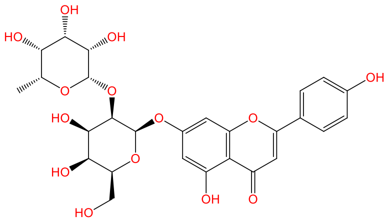	C[C@H]1O[C@@H](O[C@H]2[C@@H](Oc3cc(O)c4c(=O)cc(-c5ccc(O)cc5)oc4c3)O[C@@H](CO)[C@@H](O)[C@H]2O)[C@@H](O)[C@@H](O)[C@H]1O	-12.238
ZINC4534058	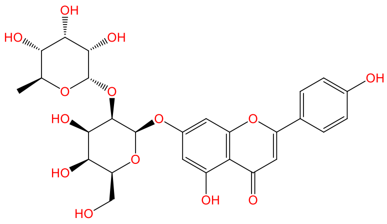	C[C@@H]1O[C@@H](O[C@H]2[C@@H](Oc3cc(O)c4c(=O)cc(-c5ccc(O)cc5)oc4c3)O[C@@H](CO)[C@@H](O)[C@H]2O)[C@@H](O)[C@@H](O)[C@H]1O	-13.697
ZINC4534059	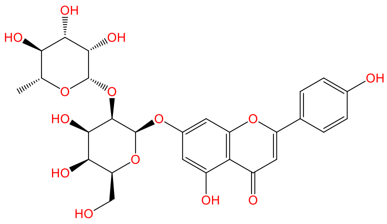	C[C@H]1O[C@@H](O[C@H]2[C@@H](Oc3cc(O)c4c(=O)cc(-c5ccc(O)cc5)oc4c3)O[C@@H](CO)[C@@H]	-14.387

		(O)[C@H]2O)[C@@H](O)[C@@H](O)[C@@H]1O	
ZINC4534060	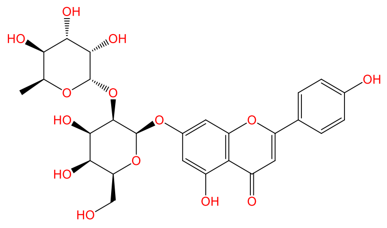	C[C@@H]1O[C@@H](O[C@H]2[C@@H](Oc3cc(O)c4c(=O)cc(-c5ccc(O)cc5)oc4c3)O[C@@H](CO)[C@@H](O)[C@H]2O)[C@@H](O)[C@@H](O)[C@@H]1O	-11.160
ZINC3978800 (Rhoifolin)	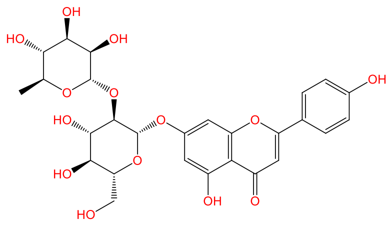	C[C@@H]1O[C@@H](O[C@H]2[C@H](Oc3cc(O)c4c(=O)cc(-c5ccc(O)cc5)oc4c3)O[C@H](CO)[C@@H](O)[C@@H]2O)[C@H](O)[C@H](O)[C@H]1O	-11.961

Supplemental Table S4

ADME characteristics of the 16 compounds from the ZINC database for natural products evaluated with the SwissADME resource.[44]

**Fraction Csp3**	The ratio of sp3 hybridized carbons over the total carbon count of the molecule
**MR**	Molecular refractivity
**TPSA**	Topological polar surface area
**iLOGP**	Efficient Description of n-Octanol/Water Partition Coefficient
**XLOGP3**	XLOGP3 predicts the logP value of a query compound by using the known logP value of a reference compound as a starting point.
**WLOGP**	Lipophilicity factor developed by Wildman and Crippen
**MLOGP**	Octanol/water partition coefficient developed by Moriguchi and Matsushita
**Silicos-IT Log P**	SILICOS-IT is the log Po/w estimation returned by executing the FILTER-IT program
**Consensus Log P**	Consensus log Po/w value is the arithmetic mean of the five predictive values above
**ESOL Log S**	Solubility, log S (calculated with the ESOL model)—Estimating Aqueous Solubility Directly from Molecular Structure
**ESOL Solubility (mg/ml)**	Estimating Aqueous Solubility Directly from Molecular Structure in mg/ml
**ESOL Solubility (mol/l)**	Estimating Aqueous Solubility Directly from Molecular Structure in mol/l
**ESOL Class**	Classification of the compound based on estimating Aqueous Solubility Directly from Molecular Structure
**Ali Log S**	In silico prediction of aqueous solubility incorporating the effect of topographical polar surface area by Ali et all.
**Ali Solubility (mg/ml)**	Aqueous Solubility developed by Ali in mg/ml
**Ali Solubility (mol/l)**	Aqueous Solubility developed by Ali in mol/l
**Ali Class**	Classification of the compound based on estimating Aqueous Solubility by Ali
**Silicos-IT LogSw**	Intrinsic water solubility estimated by Wskowwin executing the FILTER-IT program
**Silicos-IT Solubility (mg/ml)**	Intrinsic water solubility in mg/ml
**Silicos-IT Solubility (mol/l)**	Intrinsic water solubility in mol/l
**Silicos-IT class**	Classification of the compound based on the water solubility by executing the FILTER-IT program
**log Kp (cm/s)**	Value of the skin permeability coefficient

Molecule	Formula	MW	#Heavy atoms	#Aromatic heavy atoms	Fraction Csp3	#Rotatable bonds	#H-bond acceptors
**ZINC4349204 (Fortunellin)**	C28H32O14	592.55	42	16	0.46	7	14
ZINC4349029	C26H28O14	564.49	40	16	0.42	7	14
ZINC4349031	C26H28O14	564.49	40	16	0.42	7	14
ZINC4349034	C26H28O14	564.49	40	16	0.42	7	14
ZINC3983878	C26H27O14	563.48	40	16	0.42	7	14
ZINC4349207	C28H32O14	592.55	42	16	0.46	7	14
ZINC4349211	C28H32O14	592.55	42	16	0.46	7	14
ZINC4349214	C28H32O14	592.55	42	16	0.46	7	14
ZINC4349623	C27H30O14	578.52	41	16	0.44	6	14
ZINC4349627	C27H30O14	578.52	41	16	0.44	6	14
ZINC4349630	C27H30O14	578.52	41	16	0.44	6	14
ZINC4349633	C27H30O14	578.52	41	16	0.44	6	14
ZINC4534057	C27H30O14	578.52	41	16	0.44	6	14
ZINC4534058	C27H30O14	578.52	41	16	0.44	6	14
ZINC4534059	C27H30O14	578.52	41	16	0.44	6	14
ZINC4534060	C27H30O14	578.52	41	16	0.44	6	14
ZINC3978800	C27H30O14	578.52	41	16	0.44	6	14

	#H-bond donors	MR	TPSA	iLOGP	XLOGP3	WLOGP	MLOGP
**ZINC4349204 (Fortunellin)**	7	141.80	217.97	3.12	1.05	-0.80	-2.76
ZINC4349029	8	132.56	228.97	1.85	-0.36	-1.49	-3.16
ZINC4349031	8	132.56	228.97	2.11	-0.36	-1.49	-3.16
ZINC4349034	8	132.56	228.97	2.16	-0.36	-1.49	-3.16
ZINC3983878	7	129.79	231.80	2.92	-1.04	-1.10	-3.39
ZINC4349207	7	141.80	217.97	3.09	1.05	-0.80	-2.76
ZINC4349211	7	141.80	217.97	2.94	1.05	-0.80	-2.76
ZINC4349214	7	141.80	217.97	2.74	1.05	-0.80	-2.76
ZINC4349623	8	137.33	228.97	2.45	-0.16	-1.10	-2.96
ZINC4349627	8	137.33	228.97	2.03	-0.16	-1.10	-2.96
ZINC4349630	8	137.33	228.97	2.61	-0.16	-1.10	-2.96
ZINC4349633	8	137.33	228.97	2.60	-0.16	-1.10	-2.96
ZINC4534057	8	137.33	228.97	2.45	-0.16	-1.10	-2.96
ZINC4534058	8	137.33	228.97	2.63	-0.16	-1.10	-2.96
ZINC4534059	8	137.33	228.97	2.57	-0.16	-1.10	-2.96
ZINC4534060	8	137.33	228.97	2.20	-0.16	-1.10	-2.96
ZINC3978800	8	137.33	228.97	1.31	-0.16	-1.10	-2.96

	Silicos-IT Log P	Consensus Log P	ESOL Log S	ESOL Solubility (mg/ml)	ESOL Solubility (mol/l)	ESOL Class	Ali Log S
**ZINC4349204 (Fortunellin)**	-0.62	0.00	-4.00	5.99e-02	1.01e-04	Soluble	-5.22
ZINC4349029	-0.72	-0.77	-2.95	6.38e-01	1.13e-03	Soluble	-3.99
ZINC4349031	-0.72	-0.72	-2.95	6.38e-01	1.13e-03	Soluble	-3.99
ZINC4349034	-0.72	-0.71	-2.95	6.38e-01	1.13e-03	Soluble	-3.99
ZINC3983878	-0.72	-0.67	-2.51	1.73e+00	3.07e-03	Soluble	-3.34
ZINC4349207	-0.62	-0.01	-4.00	5.99e-02	1.01e-04	Soluble	-5.22
ZINC4349211	-0.62	-0.04	-4.00	5.99e-02	1.01e-04	Soluble	-5.22
ZINC4349214	-0.62	-0.08	-4.00	5.99e-02	1.01e-04	Soluble	-5.22
ZINC4349623	-1.17	-0.59	-3.22	3.50e-01	6.04e-04	Soluble	-4.19
ZINC4349627	-1.17	-0.67	-3.22	3.50e-01	6.04e-04	Soluble	-4.19
ZINC4349630	-1.17	-0.56	-3.22	3.50e-01	6.04e-04	Soluble	-4.19
ZINC4349633	-1.17	-0.56	-3.22	3.50e-01	6.04e-04	Soluble	-4.19
ZINC4534057	-1.17	-0.59	-3.22	3.50e-01	6.04e-04	Soluble	-4.19
ZINC4534058	-1.17	-0.55	-3.22	3.50e-01	6.04e-04	Soluble	-4.19
ZINC4534059	-1.17	-0.56	-3.22	3.50e-01	6.04e-04	Soluble	-4.19
ZINC4534060	-1.17	-0.64	-3.22	3.50e-01	6.04e-04	Soluble	-4.19
ZINC3978800	-1.17	-0.81	-3.22	3.50e-01	6.04e-04	Soluble	-4.19

	Ali Solubility (mg/ml)	Ali Solubility (mol/l)	Ali Class	Silicos-IT LogSw	Silicos-IT Solubility (mg/ml)	Silicos-IT Solubility (mol/l)	Silicos-IT class
**ZINC4349204 (Fortunellin)**	3.59e-03	6.05e-06	Moderately soluble	-2.16	4.06e+00	6.85e-03	Soluble
ZINC4349029	5.83e-02	1.03e-04	Soluble	-1.92	6.83e+00	1.21e-02	Soluble
ZINC4349031	5.83e-02	1.03e-04	Soluble	-1.92	6.83e+00	1.21e-02	Soluble
ZINC4349034	5.83e-02	1.03e-04	Soluble	-1.92	6.83e+00	1.21e-02	Soluble
ZINC3983878	2.58e-01	4.57e-04	Soluble	-1.92	6.82e+00	1.21e-02	Soluble
ZINC4349207	3.59e-03	6.05e-06	Moderately soluble	-2.16	4.06e+00	6.85e-03	Soluble
ZINC4349211	3.59e-03	6.05e-06	Moderately soluble	-2.16	4.06e+00	6.85e-03	Soluble
ZINC4349214	3.59e-03	6.05e-06	Moderately soluble	-2.16	4.06e+00	6.85e-03	Soluble
ZINC4349623	3.70e-02	6.40e-05	Moderately soluble	-1.48	1.92e+01	3.31e-02	Soluble
ZINC4349627	3.70e-02	6.40e-05	Moderately soluble	-1.48	1.92e+01	3.31e-02	Soluble
ZINC4349630	3.70e-02	6.40e-05	Moderately soluble	-1.48	1.92e+01	3.31e-02	Soluble
ZINC4349633	3.70e-02	6.40e-05	Moderately soluble	-1.48	1.92e+01	3.31e-02	Soluble
ZINC4534057	3.70e-02	6.40e-05	Moderately soluble	-1.48	1.92e+01	3.31e-02	Soluble
ZINC4534058	3.70e-02	6.40e-05	Moderately soluble	-1.48	1.92e+01	3.31e-02	Soluble
ZINC4534059	3.70e-02	6.40e-05	Moderately soluble	-1.48	1.92e+01	3.31e-02	Soluble
ZINC4534060	3.70e-02	6.40e-05	Moderately soluble	-1.48	1.92e+01	3.31e-02	Soluble
ZINC3978800	3.70e-02	6.40e-05	Moderately soluble	-1.48	1.92e+01	3.31e-02	Soluble

	GI absorption	BBB permeant	Pgp substrate	CYP1A2 inhibitor	CYP2C19 inhibitor	CYP2C9 inhibitor	CYP2D6 inhibitor
**ZINC4349204 (Fortunellin)**	Low	No	Yes	No	No	No	No
ZINC4349029	Low	No	Yes	No	No	No	No
ZINC4349031	Low	No	Yes	No	No	No	No
ZINC4349034	Low	No	Yes	No	No	No	No
ZINC3983878	Low	No	Yes	No	No	No	No
ZINC4349207	Low	No	Yes	No	No	No	No
ZINC4349211	Low	No	Yes	No	No	No	No
ZINC4349214	Low	No	Yes	No	No	No	No
ZINC4349623	Low	No	Yes	No	No	No	No
ZINC4349627	Low	No	Yes	No	No	No	No
ZINC4349630	Low	No	Yes	No	No	No	No
ZINC4349633	Low	No	Yes	No	No	No	No
ZINC4534057	Low	No	Yes	No	No	No	No
ZINC4534058	Low	No	Yes	No	No	No	No
ZINC4534059	Low	No	Yes	No	No	No	No
ZINC4534060	Low	No	Yes	No	No	No	No
ZINC3978800	Low	No	Yes	No	No	No	No

	CYP3A4 inhibitor	log Kp (cm/s)	Lipinski #violations	Ghose #violations	Veber #violations	Egan #violations	Muegge #violations
**ZINC4349204 (Fortunellin)**	No	-9.17	3	4	1	1	3
ZINC4349029	No	-10.00	3	3	1	1	3
ZINC4349031	No	-10.00	3	3	1	1	3
ZINC4349034	No	-10.00	3	3	1	1	3
ZINC3983878	No	-10.48	3	2	1	1	3
ZINC4349207	No	-9.17	3	4	1	1	3
ZINC4349211	No	-9.17	3	4	1	1	3
ZINC4349214	No	-9.17	3	4	1	1	3
ZINC4349623	No	-9.94	3	4	1	1	3
ZINC4349627	No	-9.94	3	4	1	1	3
ZINC4349630	No	-9.94	3	4	1	1	3
ZINC4349633	No	-9.94	3	4	1	1	3
ZINC4534057	No	-9.94	3	4	1	1	3
ZINC4534058	No	-9.94	3	4	1	1	3
ZINC4534059	No	-9.94	3	4	1	1	3
ZINC4534060	No	-9.94	3	4	1	1	3
ZINC3978800	No	-9.94	3	4	1	1	3

	Bioavailability Score	PAINS #alerts	Brenk #alerts	Leadlikeness #violations	Synthetic Accessibility		
**ZINC4349204 (Fortunellin)**	0.17	0	0	1	6.45		
ZINC4349029	0.17	0	0	1	6.08		
ZINC4349031	0.17	0	0	1	6.08		
ZINC4349034	0.17	0	0	1	6.08		
ZINC3983878	0.11	0	0	1	6.28		
ZINC4349207	0.17	0	0	1	6.45		
ZINC4349211	0.17	0	0	1	6.45		
ZINC4349214	0.17	0	0	1	6.45		
ZINC4349623	0.17	0	0	1	6.33		
ZINC4349627	0.17	0	0	1	6.33		
ZINC4349630	0.17	0	0	1	6.33		
ZINC4349633	0.17	0	0	1	6.33		
ZINC4534057	0.17	0	0	1	6.33		
ZINC4534058	0.17	0	0	1	6.33		
ZINC4534059	0.17	0	0	1	6.33		
ZINC4534060	0.17	0	0	1	6.33		
ZINC3978800	0.17	0	0	1	6.33		

Supplemental Table S5

Fully flexible binding of fortunellin and analogs PI-9 and PI30 at the fortunellin binding pocket or the active center of the 3CL-Pro, in which PI-analogs bind and inhibit the protease activity. The results are presented in changes in the Gibbs Free Energy (ΔG, in kcal/mol). See also Supplemental Figure S2 for further details.

Ligand	Binding pocket of Fortunellin ΔG (Kcal/mol)	Binding pocket of MI-09/MI-30 ΔG (Kcal/mol)
**Fortunellin**	-13.936	-11.814
**MI-09**	-16.190	-10.458
**MI-30**	-14.595	-7.862

Supplemental Figure S1

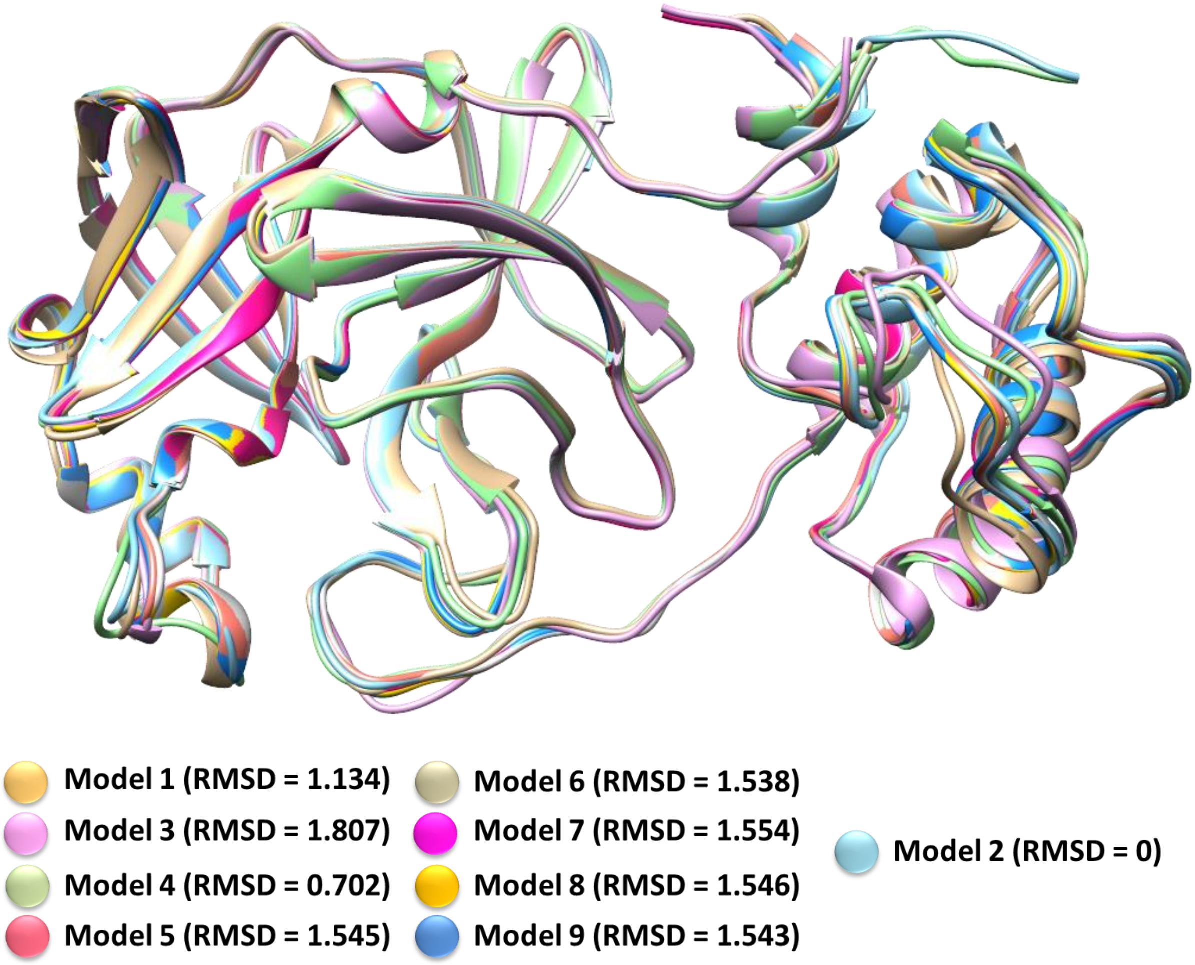

Superposition of 10 different 3CL-PRO models from the PDB database. RMSDs were calculated with the Chimera program. [45]

Supplemental Figure S2

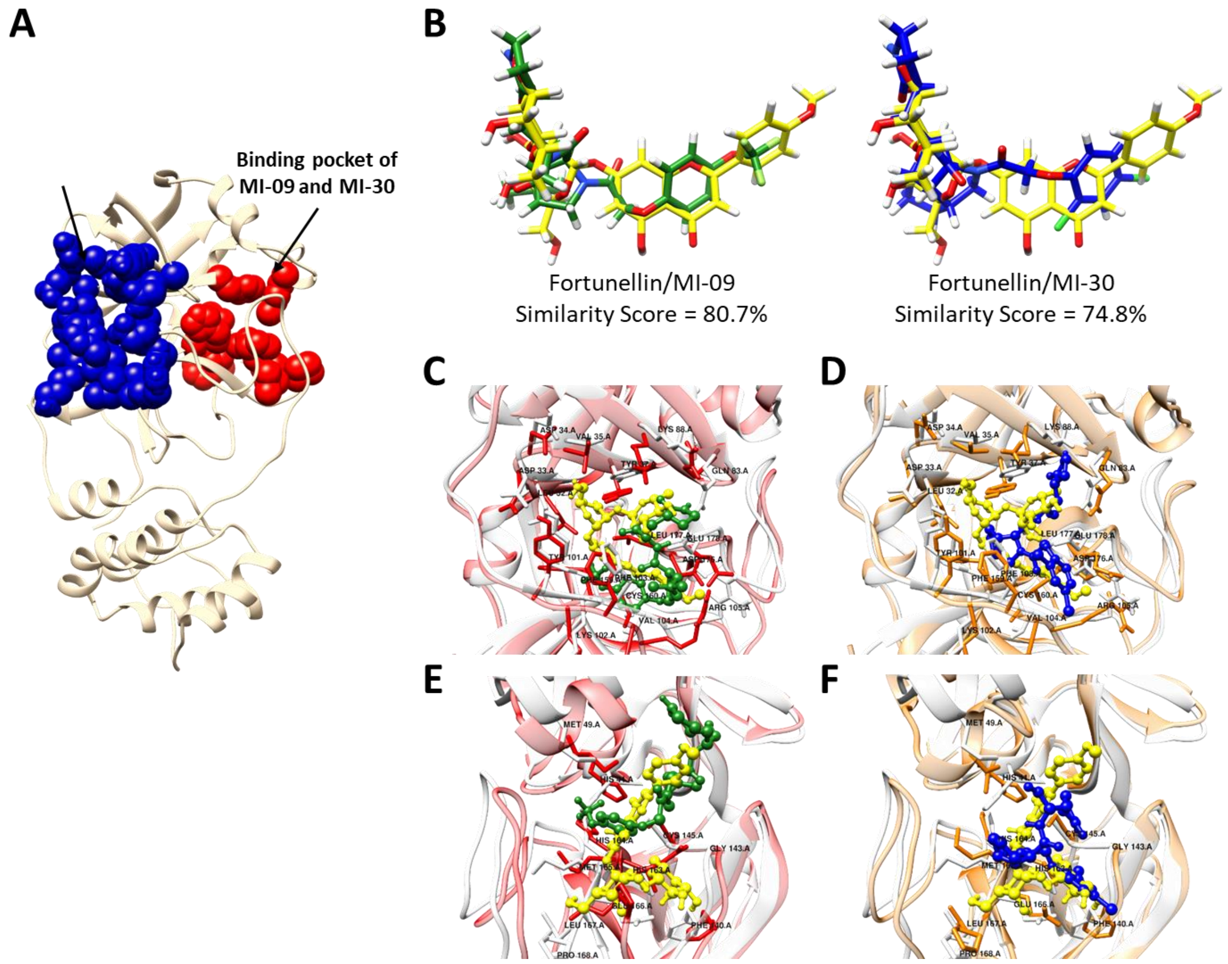

A. The binding pocket of Fortunellin (blue amino acids, Leu$_{32}$, Asp$_{33}$, Asp$_{34}$, Val$_{35}$, Tyr$_{37}$, Gln$_{83}$, Lys$_{88}$, Tyr$_{101}$, Lys$_{102}$, Phe$_{103}$, Val$_{104}$, Arg$_{105}$, Asp$_{108}$, Phe$_{159}$, Cys$_{160}$, Asp$_{176}$, Leu$_{177}$, and Glu$_{178}$) and the binding pocket of MI-09 and MI-30 (red amino acids, (Ser$_{46}$, Glu$_{47}$, Met$_{49}$, Leu$_{50}$, Ser$_{139}$, Phe$_{140}$, Leu$_{141}$, Asn$_{142}$, Gly$_{143}$ Ser$_{144}$, Cys$_{145}$, His$_{163}$, Met$_{165}$, Glu$_{166}$, His$_{172}$, and Thr$_{190}$) on the 3CL-Pro monomer (white ribbons). **B.** Alignment of Fortunellin (yellow) with MI-09 molecule (green) and MI-30 molecule (blue), performed with the in BIOVIA Discovery Studio (https://discover.3ds.com/). **C.** The binding of Fortunellin (ball and sticks yellow atoms) and MI-09 molecule (ball and sticks green atoms) in the binding pocket of Fortunellin. The conformations of amino acids when Fortunellin is binding are shown in white, and the conformations of amino acids when MI-09 is binding are shown in red. **D.** The binding of Fortunellin (ball and sticks yellow atoms) and MI-30 molecule (ball and sticks blue atoms) in the binding pocket of Fortunellin. The conformations of amino acids when fortunellin is binding are shown in white, and the conformations of amino acids when MI-30 is binding are shown in orange. **E.** The binding of Fortunellin (ball and sticks yellow atoms) and MI-09 molecule (ball and sticks green atoms) in the binding pocket of MI-09/MI-30. The conformations of amino acids when Fortunellin is binding are shown in white, and the conformations of amino acids when MI-09 is binding are shown in red. **F.** The binding of Fortunellin (ball and sticks yellow atoms) and MI-30 molecule (ball and sticks blue atoms) in the binding pocket of MI-09/MI-30. The conformations of amino acids when fortunellin is

binding are shown in white, and the conformations of amino acids when MI-30 is binding are shown in orange. Fully flexible binding was performed in the GalaxyWeb server (http://galaxy.seoklab.org/)[5–10], as described in the Methods section, above.

Supplemental Figure S3

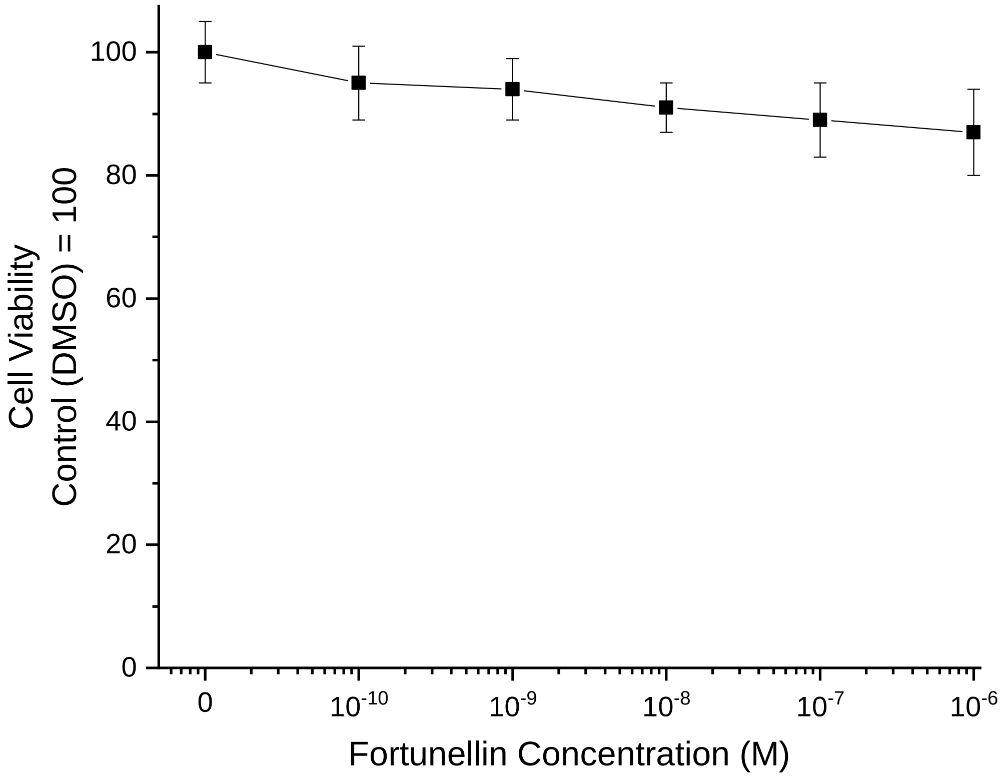

VERO cell viability after incubation for 48h with the indicated concentrations of Fortunellin diluted in DMSO. Data (obtained with the MTT assay) are presented as % of the control (DMSO) cell viability.

References

1. Arnold, K.; Bordoli, L.; Kopp, J.; Schwede, T. The SWISS-MODEL workspace: a web-based environment for protein structure homology modelling. *Bioinformatics* **2006**, *22*, 195–201, doi:10.1093/bioinformatics/bti770.

2. Berman, H.M.; Westbrook, J.; Feng, Z.; Gilliland, G.; Bhat, T.N.; Weissig, H.; Shindyalov, I.N.; Bourne, P.E. The Protein Data Bank. *Nucleic Acids Res* **2000**, *28*, 235–242.

3. Sterling, T.; Irwin, J.J. ZINC 15--Ligand Discovery for Everyone. *J Chem Inf Model* **2015**, *55*, 2324–2337, doi:10.1021/acs.jcim.5b00559.

4. O'Boyle, N.M.; Banck, M.; James, C.A.; Morley, C.; Vandermeersch, T.; Hutchison, G.R. Open Babel: An open chemical toolbox. *J Cheminform* **2011**, *3*, 33, doi:10.1186/1758-2946-3-33.

5. Baek, M.; Park, T.; Heo, L.; Park, C.; Seok, C. GalaxyHomomer: a web server for protein homo-oligomer structure prediction from a monomer sequence or structure. *Nucleic Acids Res* **2017**, *45*, W320–W324, doi:10.1093/nar/gkx246.

6. Heo, L.; Park, H.; Seok, C. GalaxyRefine: Protein structure refinement driven by side-chain repacking. *Nucleic Acids Res* **2013**, *41*, W384-8, doi:10.1093/nar/gkt458.

7. Heo, L.; Shin, W.H.; Lee, M.S.; Seok, C. GalaxySite: ligand-binding-site prediction by using molecular docking. *Nucleic Acids Res* **2014**, *42*, W210-4, doi:10.1093/nar/gku321.

8. Ko, J.; Park, H.; Heo, L.; Seok, C. GalaxyWEB server for protein structure prediction and refinement. *Nucleic Acids Res.* **2012**, *40*, W294–W297, doi:10.1093/nar/gks493.

9. Lee, G.R.; Seok, C. Galaxy7TM: flexible GPCR-ligand docking by structure refinement. *Nucleic Acids Res* **2016**, *44*, W502-6, doi:10.1093/nar/gkw360.

10. Shin, W.H.; Lee, G.R.; Heo, L.; Lee, H.; Seok, C. Prediction of Protein Structure and Interaction by GALAXY Protein Modeling Programs. *Bio Des. l Vol.2 l No.1 l March 31, 2014* **2014**, *2*, 1–11.

11. Shin, W.H.; Kim, J.K.; Kim, D.S.; Seok, C. GalaxyDock2: protein-ligand docking using beta-complex and global optimization. *J Comput Chem* **2013**, *34*, 2647–2656, doi:10.1002/jcc.23438.

12. Lee, G.R.; Won, J.; Heo, L.; Seok, C. GalaxyRefine2: simultaneous refinement of inaccurate local regions and overall protein structure. *Nucleic Acids Res* **2019**, *47*, W451–W455, doi:10.1093/nar/gkz288.

13. Lee, G.R.; Heo, L.; Seok, C. Effective protein model structure refinement by loop modeling and overall relaxation. *Proteins* **2016**, *84 Suppl 1*, 293–301, doi:10.1002/prot.24858.

14. Jin, Z.; Du, X.; Xu, Y.; Deng, Y.; Liu, M.; Zhao, Y.; Zhang, B.; Li, X.; Zhang, L.; Peng, C.; et al. Structure of Mpro from SARS-CoV-2 and discovery of its inhibitors. *Nature* **2020**, *582*, 289–293, doi:10.1038/s41586-020-2223-y.

15. Chang, G.-G. Quaternary Structure of the SARS Coronavirus Main Protease. *Mol. Biol. SARS-Coronavirus* 115–128, doi:10.1007/978-3-642-03683-5_8.

16. Dolinsky, T.J.; Nielsen, J.E.; McCammon, J.A.; Baker, N.A. PDB2PQR: an automated pipeline for the setup of Poisson–Boltzmann electrostatics calculations. *Nucleic Acids Res.* **2004**, *32*, W665–W667.

17. Mark, P.; Nilsson, L. Structure and dynamics of the TIP3P, SPC, and SPC/E water models at 298 K. *J. Phys. Chem. A* **2001**, *105*, 9954–9960.

18. Duan, Y.; Wu, C.; Chowdhury, S.; Lee, M.C.; Xiong, G.; Zhang, W.; Yang, R.; Cieplak, P.; Luo, R.; Lee, T.; et al. A point-charge force field for molecular mechanics simulations of proteins based on condensed-phase quantum mechanical calculations. *J Comput Chem* **2003**, *24*, 1999–2012, doi:10.1002/jcc.10349.

19. Sousa da Silva, A.W.; Vranken, W.F. ACPYPE - AnteChamber PYthon Parser interfacE. *BMC Res. Notes* **2012**, *5*, 367, doi:10.1186/1756-0500-5-367.

20. Petratos, K.; Gessmann, R.; Daskalakis, V.; Papadovasilaki, M.; Papanikolau, Y.; Tsigos, I.; Bouriotis, V. Structure and Dynamics of a Thermostable Alcohol Dehydrogenase from the Antarctic Psychrophile Moraxella sp. TAE123. *ACS Omega* **2020**, doi:10.1021/acsomega.0c01210.

21. Berendsen, H.J.C.; Postma, J.P.M.; van Gunsteren, W.F.; DiNola, A.; Haak, J.R. Molecular dynamics with coupling to an external bath. *J. Chem. Phys.* **1984**, *81*, 3684–3690.

22. Bussi, G.; Donadio, D.; Parrinello, M. Canonical sampling through velocity rescaling. *J. Chem. Phys.* **2007**, *126*, 14101.

23. Berendsen, H.J.C.; van der Spoel, D.; van Drunen, R. GROMACS: A message-passing parallel molecular dynamics implementation. *Comput. Phys. Commun.* **1995**, *91*, 43–56.

24. Parrinello, M.; Rahman, A. Polymorphic transitions in single crystals: A new molecular dynamics method. *J. Appl. Phys.* **1981**, *52*, 7182–7190.

25. Darden, T.; York, D.; Pedersen, L. Particle mesh Ewald: An N· log (N) method for Ewald sums in large systems. *J. Chem. Phys.* **1993**, *98*, 10089–10092.

26. Yeh, I.-C.; Berkowitz, M.L. Ewald summation for systems with slab geometry. *J. Chem. Phys.* **1999**, *111*, 3155–3162.

27. Hess, B.; Bekker, H.; Berendsen, H.J.C.; Fraaije, J.G.E.M. LINCS: a linear constraint solver for molecular simulations. *J. Comput. Chem.* **1997**, *18*, 1463–1472.

28. Pande, V.S.; Beauchamp, K.; Bowman, G.R. Everything you wanted to know about Markov State Models but were afraid to ask. *Methods* **2010**, *52*, 99–105, doi:https://doi.org/10.1016/j.ymeth.2010.06.002.

29. Prinz, J.-H.; Wu, H.; Sarich, M.; Keller, B.; Senne, M.; Held, M.; Chodera, J.D.; Schütte, C.; Noé, F. Markov models of molecular kinetics: Generation and validation. *J. Chem. Phys.* **2011**, *134*, 174105, doi:10.1063/1.3565032.

30. Chodera, J.D.; Noé, F. Markov state models of biomolecular conformational dynamics. *Curr. Opin. Struct. Biol.* **2014**, *25*, 135–144, doi:https://doi.org/10.1016/j.sbi.2014.04.002.

31. Plattner, N.; Noé, F. Protein conformational plasticity and complex ligand-binding kinetics explored by atomistic simulations and Markov models. *Nat. Commun.* **2015**, *6*, 7653, doi:10.1038/ncomms8653.

32. Plattner, N.; Doerr, S.; De Fabritiis, G.; Noé, F. Complete protein–protein association kinetics in atomic detail revealed by molecular dynamics simulations and Markov modelling. *Nat Chem* **2017**, *advance on*, doi:10.1038/nchem.2785http://www.nature.com/nchem/journal/vaop/ncurrent/abs/nchem.2785.html#supplementary-information.

33. Voelz, V.A.; Bowman, G.R.; Beauchamp, K.; Pande, V.S. Molecular Simulation of ab Initio Protein Folding for a Millisecond Folder NTL9(1−39). *J. Am. Chem. Soc.* **2010**, *132*, 1526–1528, doi:10.1021/ja9090353.

34. Durrant, J.D.; Kochanek, S.E.; Casalino, L.; Ieong, P.U.; Dommer, A.C.; Amaro, R.E. Mesoscale All-Atom Influenza Virus Simulations Suggest New Substrate Binding Mechanism. *ACS Cent. Sci.* **2020**, *6*, 189–196, doi:10.1021/acscentsci.9b01071.

35. Scherer, M.K.; Trendelkamp-Schroer, B.; Paul, F.; Pérez-Hernández, G.; Hoffmann, M.; Plattner, N.; Wehmeyer, C.; Prinz, J.-H.; Noé, F. PyEMMA 2: A Software Package for Estimation, Validation, and Analysis of Markov Models. *J. Chem. Theory Comput.* **2015**, *11*, 5525–5542, doi:10.1021/acs.jctc.5b00743.

36. Noé, F. Probability distributions of molecular observables computed from Markov models. *J. Chem. Phys.* **2008**, *128*, 244103, doi:10.1063/1.2916718.

37. Trendelkamp-Schroer, B.; Wu, H.; Paul, F.; Noé, F. Estimation and uncertainty of reversible Markov models. *J. Chem. Phys.* **2015**, *143*, 174101, doi:10.1063/1.4934536.

38. Wu, H.; Noé, F. Variational Approach for Learning Markov Processes from Time Series Data. *J. Nonlinear Sci.* **2020**, *30*, 23–66, doi:10.1007/s00332-019-09567-y.

39. Sutto, L.; Marsili, S.; Gervasio, F.L. New advances in metadynamics. *Wiley Interdiscip. Rev. Comput. Mol. Sci.* **2012**, *2*, 771–779.

40. Barducci, A.; Bussi, G.; Parrinello, M. Well-tempered metadynamics: A smoothly converging and tunable free-energy method. *Phys. Rev. Lett.* **2008**, *100*, 20603.

41. Bonomi, M.; Parrinello, M. Enhanced sampling in the well-tempered ensemble. *Phys. Rev. Lett.* **2010**, *104*, 190601.

42. Bussi, G.; Gervasio, F.L.; Laio, A.; Parrinello, M. Free-Energy Landscape for β Hairpin Folding from Combined Parallel Tempering and Metadynamics. *J. Am. Chem. Soc.* **2006**, *128*, 13435–13441, doi:10.1021/ja062463w.

43. Tribello, G.A.; Bonomi, M.; Branduardi, D.; Camilloni, C.; Bussi, G. PLUMED 2: New feathers for an old bird. *Comput. Phys. Commun.* **2014**, *185*, 604–613.

44. Daina, A.; Michielin, O.; Zoete, V. SwissADME: a free web tool to evaluate pharmacokinetics, drug-likeness and medicinal chemistry friendliness of small molecules. *Sci Rep* **2017**, *7*, 42717, doi:10.1038/srep42717.

45. Pettersen, E.F.; Goddard, T.D.; Huang, C.C.; Couch, G.S.; Greenblatt, D.M.; Meng, E.C.; Ferrin, T.E. UCSF Chimera--a visualization system for exploratory research and analysis. *J Comput Chem* **2004**, *25*, 1605–1612, doi:10.1002/jcc.20084.
